# Methodological standards for body composition assessment—an expert-endorsed guide for research and clinical applications: bioimpedance, dual-energy X-ray absorptiometry, computerized tomography, and ultrasound methods

**DOI:** 10.1016/j.ajcnut.2026.101283

**Published:** 2026-03-19

**Authors:** Carla M Prado, M Cristina Gonzalez, Kristina Norman, Camila E Orsso, Rocco Barazzoni, Tommy Cederholm, Charlene Compher, Gordon L Jensen, Takashi Abe, Thiago Gonzalez Barbosa-Silva, Jonathan P Bennett, Owen T Carmichael, Carrie P Earthman, William J Evans, David A Fields, Laurence Genton, Houchun Harry Hu, Murat Kara, Jennifer L Miles-Chan, Marina Mourtzakis, Michael T Paris, Stany Perkisas, Luís B Sardinha, John A Shepherd, Mario Siervo, Boyd JG Strauss, Yosuke Yamada, Shankuan Zhu, Steven B Heymsfield

**Affiliations:** 1 Department of Agricultural, Food and Nutritional Science, Faculty of Agricultural, Life and Environmental Sciences, University of Alberta, Edmonton, AB, Canada; 2 Postgraduate Program in Nutrition and Food, Federal University of Pelotas, Pelotas, Rio Grande do Sul, Brazil; 3 Department of Geriatrics, Charité Universitätsmedizin Berlin, Berlin, Germany; 4 Department of Nutrition and Gerontology, German Institute of Human Nutrition Potsdam-Rebrücke, Nuthetal, Germany; 5 Department of Medical, Surgical and Health Sciences, University of Trieste, Ospedale di Cattinara, Trieste, Italy; 6 Department of Public Health and Caring Sciences, Clinical Nutrition and Metabolism, Uppsala University, Uppsala, Sweden; 7 Theme Inflammation and Ageing, Karolinska University Hospital, Stockholm, Sweden; 8 Department of Biobehavioral Health Science, University of Pennsylvania, School of Nursing, Philadelphia, PA, United States; 9 Dean’s Office and Department of Medicine, University of Vermont Larner College of Medicine, Burlington, VT, United States; 10 Institute of Health and Sports Science & Medicine, Graduate School of Health and Sports Science, Juntendo University, Chiba, Japan; 11 Department of General Surgery, Faculty of Medicine, Federal University of Pelotas, Pelotas, Rio Grande do Sul, Brazil; 12 Department of Epidemiology, University of Hawaii Cancer Center, Honolulu, HI, United States; 13 Pennington Biomedical Research Center, Louisiana State University System, Baton Rouge, LA, United States; 14 Department of Health Behavior and Nutrition Sciences, College of Health Sciences, University of Delaware, Newark, DE, United States; 15 Department of Nutritional Sciences and Toxicology, University of California Berkeley, Berkeley, CA, United States; 16 Division of Geriatrics, Duke University School of Medicine, Durham, NC, United States; 17 Department of Pediatrics, Section of Diabetes and Endocrinology, University of Oklahoma Health Sciences Center, Oklahoma City, OK, United States; 18 Clinical Nutrition, Department of Medicine, Geneva University Hospitals, Geneva, Switzerland; 19 Department of Radiology, Mayo Clinic, Jacksonville, FL, United States; 20 Department of Physical Medicine and Rehabilitation, Hacettepe University Medical School, Ankara, Turkey; 21 Human Nutrition Unit, School of Biological Sciences, University of Auckland, Auckland, New Zealand; 22 Department of Kinesiology and Health Sciences, Faculty of Health, University of Waterloo, Waterloo, ON, Canada; 23 School of Kinesiology and Health Science, York University, TO, Canada; 24 University Center for Geriatrics, University of Antwerp/ZNA (Ziekenhuis aan de Stroom), Antwerp, Belgium; 25 Exercise and Health Laboratory, CIPER, Faculdade de Motricidade Humana, Universidade de Lisboa, Cruz Quebrada, Portugal; 26 School of Population Health, Curtin University, Perth, Western Australia, Australia; 27 Curtin Dementia Centre of Excellence, enAble Institute, Curtin University, Perth, Western Australia, Australia; 28 Department of Medicine, School of Clinical Sciences, Monash University, Melbourne, Victoria, Australia; 29 Division of Diabetes, Endocrinology and Gastroenterology, School of Medical Sciences, Faculty of Biology, Medicine and Health, The University of Manchester, Manchester, United Kingdom; 30 Graduate School of Biomedical Engineering, Tohoku University, Sendai, Japan; 31 Department of Medicine and Science in Sports and Exercise, Graduate School of Medicine, Tohoku University, Sendai, Japan; 32 Chronic Disease Research Institute, The Children’s Hospital, and National Clinical Research Center for Child Health, School of Public Health, School of Medicine, Zhejiang University, Hangzhou, China; 33 Department of Nutrition and Food Hygiene, School of Public Health, School of Medicine, Zhejiang University, Hangzhou, China

**Keywords:** body composition, methods, terminology, validity, measurement protocol, adipose tissue, fat mass, skeletal muscle, fat-free mass, lean soft tissue

## Abstract

The assessment of body composition has long been a fundamental component of research and is gaining increasing adoption in clinical practice. This growing interest has drawn new professionals to the field and increased emphasis on its clinical relevance and applications. However, the diversity of assessment techniques and inconsistent terminology create challenges, highlighting the urgent need for harmonized approaches across research and healthcare settings. Commonly employed methods include bioelectrical impedance approaches, dual-energy X-ray absorptiometry, and computerized tomography, with ultrasound emerging as an increasingly prominent tool. These methods are featured in guidelines for diagnosing conditions such as low muscle mass, malnutrition, sarcopenia, and sarcopenic obesity, among others. This second narrative review in a series, developed by an international panel of experts, focuses on these widely accessible assessment tools that align with clinical recommendations. It presents foundational knowledge, discusses validity and reliability considerations, and offers practical advice on terminology, measurement protocols, data interpretation, and longitudinal monitoring. The report also addresses current limitations and identifies areas needing further research. Our goal is to provide clear, evidence-based guidance that is useful for both experienced practitioners and those newly engaging with body composition assessment. We urge organizations, journals, and stakeholders across the body composition field to adopt the proposed principles and standards to support consistency, transparency, and scientific rigor in both research and clinical care.

## Introduction

Body composition is well established in research and is gaining momentum as a valuable tool in clinical practice [1,2], particularly in light of recent developments in the definition of clinical obesity, and obesity medications and their reported effects on body composition [3,4]. These developments have attracted new researchers and clinicians, sparking growing interest in the clinical relevance of body composition changes to health outcomes. Consequently, there is a pressing need for greater clarity and standardization in how body composition is assessed, interpreted, and applied across both research and clinical settings.

Evaluating body composition provides information on nutrition and health status, supports personalized nutrition interventions, informs treatment decisions, and quantifies predictors of functional decline, disease progression, and adverse clinical outcomes [5,6]. Widely available body composition methods include bioelectrical impedance approaches, dual-energy X-ray absorptiometry (DXA), computerized tomography (CT), and ultrasound (US). These methods are recommended in clinical guidelines that enumerate body composition criteria for diagnosing low muscle mass, malnutrition, sarcopenia, and sarcopenic obesity [[7], [8], [9], [10], [11]].

Body composition methods differ across multiple dimensions, posing challenges for their application [2,12,13]. Methods vary in technology, body components assessed, level of expertise required for use, validity, cost, portability, and safety. Each method also involves specific equipment, protocols, and data presentation formats, further adding to this complexity. The multiple dimensions of each method can present meaningful challenges for researchers and clinicians in determining what to measure, how to measure it, and when assessments should occur [14], often resulting in suboptimal use of body composition methods and acquired data.

To help clarify key concepts, promote consistent scientific terminology, and provide practical guidance on the use and interpretation of body composition information, we convened an international working group of selected experts in the field [15]. In this second publication of our series, we focus on widely available body composition methods aligned with proposed clinical guidelines for body composition assessment, including bioelectrical impedance technologies, DXA, CT, and US [[7], [8], [9], [10], [11]]. Other methods such as MRI are less commonly utilized and will therefore be addressed in a subsequent publication. For each method, we review foundational principles and provide practical recommendations regarding terminology, assessment procedures, data presentation, applications across specific populations, and comparisons with other methods. Key concepts such as method validity, reliability (precision), and minimal detectable change (MDC) are discussed to provide clinically relevant interpretation of body composition changes. Limitations and existing research gaps are also highlighted. Our aim is to make this information, grounded in both scientific evidence and expert consensus, accessible and relevant to experienced professionals and those new to the field. We encourage relevant organizations, journals, and stakeholders in the body composition field to endorse the principles in this report and the standardized terminology proposed previously [15].

## Methods

We followed a structured outline to collect data on each body composition method, covering the following topics: core principles, level and components assessed/estimated, proposed standardized terminology, reliability and validity, applicability for longitudinal monitoring, clinical considerations, common operational errors, standards for assessment procedures and data reporting, cross-comparison between devices and software, a summary of pros and cons, and recommendations to enhance practical utility and inform industry development. Working group members were assigned to subgroups based on expertise to complete these sections with supporting evidence. Contributions were compiled into a full draft, circulated for group-wide feedback. Any disagreements identified during review were resolved through subgroup discussions and further rounds of review. To maximize usability, sections were designed to stand alone so that readers can directly consult the body composition method of interest; as a result, some repetition may be found across sections. Summary boxes are included to support appropriate application, interpretation, and reporting of body composition assessment by synthesizing key considerations, best practices, limitations, and research gaps.

On the basis of this report and the standardized terminology proposed in the preceding publication [15], we created a Body Composition Terminology & Reporting Checklist to promote consistency and standardization across both research and clinical practice (Supplemental Information 1). Readers are encouraged to refer to the first publication in this series [15] and Supplemental Table 1 for a glossary of body composition terms. Key terminology for measurement validity is presented in Supplemental Table 2, and a decision tree for selecting assessment methods is provided in Supplemental Figure 1.

## Bioimpedance Methods

Bioelectrical impedance (herein referred to as “bioimpedance”) methods measure electrical current conductance properties of body tissues. Briefly, these methods measure the body’s opposition (i.e., impedance, Z, measured in ohms) to the flow of alternating currents transmitted through tissues at ≥1 frequencies. Resistance and reactance are the Cartesian components of the impedance vector, which can also be expressed in its polar form as a modulus (|Z|, the impedance) and a phase angle (commonly abbreviated to PhA) [16]. Impedance in an alternating current electrical circuit reflects the combined effects of 2 electrical properties: resistance and reactance.

Resistance is the component of impedance that arises from the conductive properties of body tissues. Tissues with low electrolyte-containing fluid content, such as adipose tissue (AT) and bone, are poor conductors of electricity and therefore exhibit high resistance and impedance. In contrast, tissues with high fluid content, such as skeletal muscle and other major organs, such as the brain, kidneys, and lungs, conduct electricity more effectively and present lower resistance and impedance. Among these organs, skeletal muscle is the main determinant of bioimpedance, as it comprises the largest hydrated tissue compartment, contains substantial intracellular water (ICW), and is widely distributed throughout the body. Regardless of tissue composition, whole-body resistance primarily reflects the volume and distribution of electrolyte-containing fluids that form the conductive pathways for electrical current. Bioimpedance therefore reflects body water volume and distribution, with estimates of AT or fat mass (FM) derived indirectly through prediction equations or as residual components of body mass after accounting for conductive fluid compartments. However, bioimpedance is frequently described in overly simplistic terms, which can obscure its strong dependence on body water volume and distribution and lead to misconceptions about what the method truly measures.

Reactance reflects the capacitive properties of cell membranes. Essentially, cell membranes act like tiny storage units for electricity (capacitors). When an alternating current flow through the body, these membranes briefly hold and slow down the electrical signal before letting it pass through. This delay is quantified as reactance and is interpreted to reflect the integrity and quantity of body cells. Evidence from a study in athletes demonstrates that reductions in resistance, reactance, and PhA are proportional to the severity of muscle injury, with decreases in reactance and PhA attributed to disruption of cellular membrane integrity [17]. Thus, reactance serves as an indicator of cellularity and membrane functionality, with higher values generally indicating greater cell mass and preserved membrane integrity.

Impedance, resistance, and reactance are all evaluated by the various types of bioimpedance methods reviewed in the following sections. The arctangent of the ratio between reactance and resistance is termed PhA; although not a direct measure of body composition, lower PhA values are an indicator of poor cellular health [18] and PhA is positively associated with fat-free mass (FFM) [19]. However, despite its potential clinical relevance, PhA should not be interpreted without ensuring standardized measurement conditions and appropriate reference values.

**FIGURE 1:**
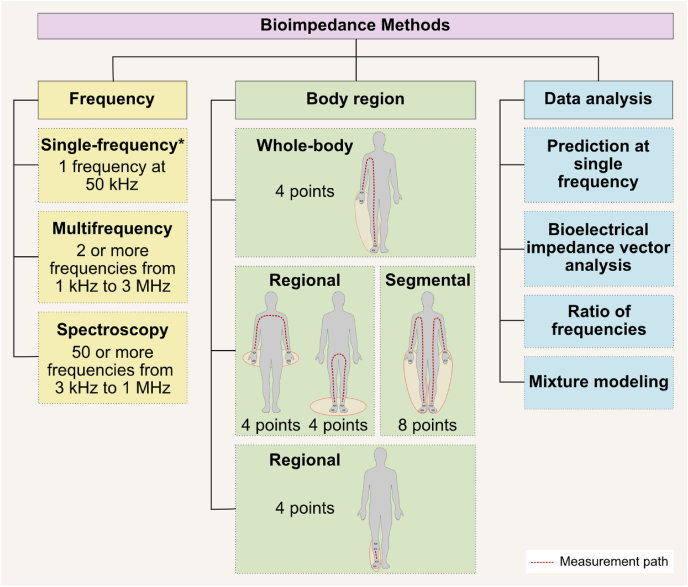
Bioimpedance methods differ based on frequency, the body region assessed, and the approaches used for data analysis. “Points” refers to points of contact (i.e., location where the electrodes are placed in the body). Images retrieved from smart.servier.com. ∗Single-frequency bioelectrical impedance analysis technology can be further classified as nonphase sensitive and phase sensitive.

Three main classes of bioimpedance devices are available: single-frequency bioelectrical impedance analysis (SF-BIA); multifrequency bioelectrical impedance analysis (MF-BIA); and bioimpedance spectroscopy (BIS) [12] (Figure 1). In SF-BIA an alternating electric current (≤0.8 mA) passes through the body at a single frequency (50 kHz), whereas MF-BIA devices include ≥2 (typically ≤8) frequencies ranging from 1 kHz up to 1 MHz, with some devices utilizing ≤3 MHz [20]. BIS devices, which employ a modified bioelectrical impedance analysis (BIA) approach, apply the alternating electric current over a spectrum of frequencies, ≤250 in some models, in the range of ∼3 kHz up to 1.2 MHz.

Both MF-BIA and BIS devices exploit the principle that low-frequency currents pass traverse primarily the extracellular water (ECW) compartment, whereas high-frequency currents penetrate both ECW and ICW, thereby reflecting total body water (TBW). MF-BIA devices estimate ICW indirectly as the difference between estimates of TBW and ECW, whereas BIS devices estimate ICW and ECW separately using complex modeling algorithms. Despite methodological differences, both MF-BIA and BIS may provide enhanced insight into fluid distribution and body compartmentalization, which may be particularly useful in certain populations. Because SF-BIA and MF-BIA differ fundamentally from BIS, we recommend using the more general term “bioimpedance methods” when referring to any of these 3 classes of bioimpedance devices, and “BIA” when referring to SF-BIA and/or MF-BIA only.

Box 1 outlines key considerations for bioimpedance-based body composition assessment. Table 1 summarizes the technical and practical characteristics of this method, whereas best-use scenarios and common pitfalls are presented in Supplemental Boxes 1 and 2, respectively. Common myths are summarized in Supplemental Table 3, practices to avoid in Supplemental Box 3, and key research gaps in Supplemental Box 4.

BOX 1 **Key points on body composition assessment using bioimpedance methods**

• Measure tissue electrical impedance/conductance properties rather than body components directly. Single-frequency and multifrequency bioelectrical impedance analysis devices rely on population- and device-specific prediction equations, whereas bioimpedance spectroscopy uses impedance across a broad range of frequencies with biophysical modeling.
• Raw measurements (e.g., reactance, resistance, impedance vectors) should be considered primary data, whereas derived body composition estimates should be regarded as model-based outputs that depend on underlying assumptions.
• Accuracy depends on the device, software, and prediction equations, and how closely the individual matches the characteristics of the equation development sample; individual-level error can be substantial.
• Measurements are highly repeatable under standardized conditions, making them useful for longitudinal monitoring when time of day, body position, hydration status, fasting status, and environment are controlled.
• Different configurations exist, and physiological/behavioral factors (e.g., disease-related fluid shifts, edema/ascites, recent food or fluid intake, exercise, and circadian variation) can alter assumptions and affect accuracy/reliability, and interpretation.
• Report body composition estimates using standardized terminology, aligned with the reference method used for equation development.

Alt-text: BOX 1

**TABLE 1:** Summary of technical and practical characteristics of body composition assessment using bioimpedance methods.

Body components	Precision/accuracy	Key factors affecting precision/accuracy	Practical considerations	Contraindications
SF-BIA: FM, FFM, skeletal muscle, TBW MF-BIA: FM, FFM, skeletal muscle, TBW, ECW, ICW (estimated from the difference between TBW and ECW) BIS: ECW, ICW, BCM	High precision Low to moderate accuracy	Device: instrument variability and electrode characteristics (quality, size, type, and placement) Operator: electrode placement Prediction equation (SF-BIA, MF-BIA): reference standard, population, and device for developing/validating equations Subject: age, ethnicity, body shape and adiposity, hydration and fluid changes, circadian variations, skin temperature, postural changes, meal ingestion, silicone breast implants	Portable and stationary systems options No subject size limitations, except for weight restrictions in standing systems No radiation exposure (0 mSv) ∼2–5 min for measurement, plus preparation time Low to moderate device cost; low per-test cost	Altered hydration or high adiposity may reduce accuracy Although generally safe, this method may not be used in individuals with pacemakers or other implantable cardiac devices, in accordance with local regulations

Abbreviations: BCM, body cell mass; BIS, bioimpedance spectroscopy; ECW, extracellular water; FFM, fat-free mass; FM, fat mass; ICW, intracellular water; MF-BIA, multifrequency bioelectrical impedance analysis; SF-BIA, single-frequency bioelectrical impedance analysis; TBW, total body water.

### Principles and terminology of bioimpedance methods

#### Bioelectrical impedance analysis

**FIGURE 2:**
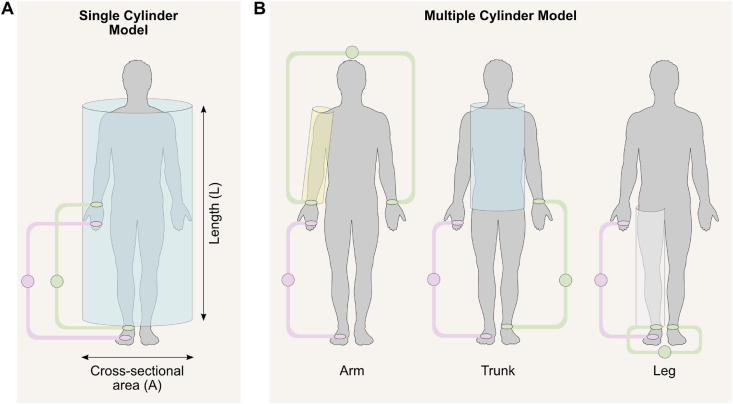
Schematic of the human body modeled as cylindrical conductors for bioimpedance analysis [16]. Images retrieved from smart.servier.com. (A) Whole-body bioimpedance analysis. The body is modeled as a single uniform cylinder. The principle of resistance (*R*), the real component of impedance (Z), is that it is proportional to the conductor’s length (L) and inversely proportional to its cross-sectional area (A), expressed as *R* = ρ × (L / A), where ρ is the resistivity of biological tissue. Using body length and measured resistance, the volume of conductive tissue can be estimated (V = A × L), which can be rearranged to V = L^2^ / R. Substituting height (Ht) for body length and assuming a constant ρ, the ratio Ht^2^ / *R* serves as an index of conductive volume and is sometimes referred to as the impedance index. This index strongly correlates with TBW and FFM, as validated by dilution methods, hydrostatic weighing, and dual-energy X-ray absorptiometry. Given that ∼73% of FFM is composed of water, TBW can be used to estimate FFM. Subsequently, fat mass is derived as the difference between total body weight and FFM. (B) Segmental bioimpedance analysis. The body is represented as 5 cylinders corresponding to the limbs and trunk. Colored circuits show the current-injecting (purple) and voltage-sensing (green) electrodes. The figure illustrates example measurements for the right arm, trunk, and right leg, enabling regional estimates of resistance and derived body composition. FFM, fat-free mass; TBW, total body water.

The physical principles of SF-BIA and MF-BIA are shown in Figure 2. A cylinder model is used to relate impedance to body geometry and conductive tissue properties for estimating TBW [16,21]. However, the body is not a uniform cylinder with a constant conductivity, and therefore, correction factors are needed to account for individual variation in moderating factors such as age, hydration status, and/or body shape. These factors are typically incorporated into population-specific prediction equations used to estimate BIA-derived body composition components, such as TBW and FFM or other muscle-related components [21,22]. To account for variation in body composition across body regions, segmental BIA systems evaluate impedance or its components, resistance and reactance, across the trunk, arms, and legs, treating each one as a separate cylinder model with distinct conductive properties [16]. BIA devices can be classified as either phase-sensitive or non–phase-sensitive based on their electronic design. Phase-sensitive BIA devices measure in real time both the magnitude of impedance and the phase shift between the applied current and the detected voltage, known as the PhA. From these direct measurements, resistance and reactance can be mathematically derived using standard trigonometric equations, and body components are then estimated using regression equations that incorporate multiple biophysical determinants. In contrast, non–phase-sensitive SF-BIA devices measure only the magnitude of the impedance, without measuring the phase shift. As a result, they cannot provide direct measurements of PhA or allow calculation of resistance and reactance. Non–phase-sensitive MF-BIA devices can provide algorithm-based estimates of PhA, but their body composition outputs remain limited to regression models derived from impedance values across multiple frequencies or impedance adjusted for height.

Overall, BIA devices do not directly measure body components but instead use population- and device-specific prediction equations to estimate body composition [21,22]. The prediction equations are derived from validation studies against reference methods such as DXA, MRI, or deuterium dilution (for TBW) and include resistance, reactance, and impedance ratios of specific body segments, along with additional variables such as age, sex, weight, height, waist circumference, and physical activity level [23]. Prediction equations are device-specific, because the raw BIA measurements are not interchangeable across different devices (e.g., electrodes and their configuration may vary), and component prediction formulas are typically developed on a proprietary device, with manufacturers not disclosing details of the evaluated samples, the actual prediction formulas and their methods of validation [[24], [25], [26]]. Bioimpedance equations are also population-specific, as limb shape, muscularity, and body proportions can differ across race and ethnic groups, whereas age and sex are also contributors to models [27]. Bioimpedance prediction equations, when developed carefully and on a large and well-defined sample, perform well estimating body composition in individuals who are well hydrated and who are within the range of characteristics of the prediction equation development sample. However, variability in estimates can be large at the individual level [12]. For example, when SF-BIA–predicted skeletal muscle mass (SMM) from an equation reported by Janssen et al. [28] is compared with MRI-measured SMM, the mean difference (group difference) is −0.02 kg. However, at the individual level, this difference can vary from –5.3 to + 5.3 kg. Although the mean difference is close to 0 for group-level assessment, the variability may be unacceptable in clinical practice when an individual patient is being assessed and then tracked over time [28].

Bioimpedance prediction equations are usually developed on small samples that are not ethnically diverse. The developed prediction formulas are usually designed to give estimates of total body and regional FM (or percent fat mass, %FM), FFM or muscle-related compartments [such as SMM, appendicular lean soft tissue (ALST)], and TBW and ECW. Alternatively, researchers frequently publish new empirical prediction equations based on data collected across multiple studies with greater sample diversity. In either case, selected ground-truth reference methods used in model development include DXA, MRI, air-displacement plethysmography (ADP), stable isotope dilution, and markers of ECW (such as bromide). Reference methods for prediction equation development also include 3- and 4-component molecular-level models [e.g., measured TBW by deuterium dilution, bone mineral content (BMC) from DXA, and body volume from ADP are used to derive the 4 components]. As detailed in our previous publication [15], standard terminology from the reference method should be used for BIA body composition estimates. Furthermore, some manufacturers report proprietary indices such as “visceral fat rating,” “metabolic age,” or “body type.” Although these metrics may serve as engagement tools, they should not be interpreted as direct measures of visceral AT (VAT) nor considered comparable with imaging-derived VAT. These indices are typically derived from anthropometric and impedance variables, and their underlying algorithms and validation processes lack transparency.

#### Bioimpedance spectroscopy

BIS builds on the same principles of SF-BIA and MF-BIA by measuring impedance across a broad range of frequencies. This enables BIS to capture the frequency-dependent electrical behavior of biological tissues [21]. At low frequencies, an electrical current conduct primarily through the ECW compartment, whereas at high frequencies, it penetrates cell membranes and flows through all fluid compartments, reflecting both ECW and ICW (thus, TBW). These impedance values are interpreted using biophysical modeling, based on Hanai mixture theory, which represents the body as a suspension of conductive (fluids) and nonconductive (cells) components to estimate fluid compartment volumes [29].

To derive estimates from the impedance spectrum, BIS applies Cole-Cole modeling, fitting the impedance values into a semicircular arc plotted in the complex plane, simply referred to as the Cole plot. The intercept of this arc corresponds to resistance at zero (R_0_) and infinite frequency (R∞); these resistance values are then put into the mixture-based equations, which account for cellular volume fractions and the electrical contrasts between intracellular and extracellular compartments, enabling fluid volume estimation without reliance on empirical regression equations [21,30].

Some advanced MF-BIA devices incorporate Cole modeling to estimate ECW and TBW (and ICW by subtraction); however, because of the proprietary nature of their prediction algorithms, it is often unclear whether they apply full-spectrum modeling or simply use select frequencies to approximate these compartments [31]. The combination of biophysical modeling and full-spectrum impedance measurement distinguishes BIS from MF-BIA approaches. Although BIS is theoretically expected to provide greater accuracy in body composition estimation, studies have reported comparable performance between BIS and MF-BIA devices, with some MF-BIA approaches demonstrating superior results in certain contexts [32]. Discrepancies such as these may be observed for many potential reasons. Notably, all bioimpedance approaches (SF-BIA, MF-BIA, and BIS) rely on underlying assumptions and fixed constants that may be violated in certain individuals, particularly those with excessive adiposity. Consequently, performance depends on the specific compartment being estimated and the appropriateness of the reference method used for comparison. Importantly, all bioimpedance techniques are calibrated against reference methods to obtain estimates of body compartments; therefore, meaningful cross-validation requires careful selection of the reference method most suitable for evaluating the compartment of interest.

#### Bioimpedance measurement configurations

Multiple measurement configurations exist across bioimpedance technologies, each with implications for accuracy, assumptions, and interpretation of body composition estimates. Although frequency selection influences sensitivity to fluid compartments, the electrode configuration and anatomical coverage (Figure 1) are equally critical. This section describes how electrode placement and current pathways define both the measurement scope (whole-body compared with regional) and the type of impedance data acquired (single path compared with segmental).

Traditional single-frequency bioimpedance systems employ a tetrapolar (4-electrode) configuration, with electrodes positioned at the wrist and ankle, to measure frequency-dependent impedance across the entire body. These setups are considered whole-body measurements and are widely used in clinical and research settings. Technological advances have led to more compact devices that use hand-to-hand or foot-to-foot pathways, often with 2 sets of contact points [33,34]. These systems measure a limited region of the body (e.g., upper or lower body) and then estimate whole-body composition using prediction equations, introducing potential bias when fat distribution is uneven [35]. Although validated in many populations, such configurations require users to recognize the assumptions and limitations inherent in extrapolating regional data to the whole body.

In contrast, systems with 8-electrode configurations apply current and voltage sensors at each hand and foot, enabling independent impedance measurements for the limbs and trunk. This allows for true segmental bioimpedance analysis using multiple current pathways [36]. Although these devices can theoretically measure all 5 major segments to estimate whole-body values, they should not be conflated with traditional whole-body systems. Some 4-electrode devices are described as “whole-body segmental” because of their estimated regional outputs, but they do not perform independent segmental measurements. In the case of certain 4-electrode systems, electrodes are manually applied to a single region (e.g., calf or thigh) to isolate segment-specific impedance, a method referred to as localized bioimpedance, a configuration distinct from both single-path and segmental systems. To maintain consistency across studies, researchers should clearly specify both the anatomical scope and electrode configuration, and the type of bioimpedance system used [37]. Mislabeling whole-body or single-path systems as segmental, or failing to clarify the measurement region, can lead to misinterpretation and reduce reproducibility across the literature.

### Reliability and validity of bioimpedance methods

Bioimpedance measurements are highly repeatable when using the same device and following a standardized procedure, with intraclass correlation coefficients (ICCs) ≥0.99 and 1%−2% variability between repeated impedance measurements [12,21,38]. The precision of repeated %FM measurements using both standing and supine MF-BIA devices is also high [coefficient of variation (CV) <1%] [39]. Furthermore, a high ICC was observed for repeated measurements of %FM, FM, and FFM (≥0.98) using MF-BIA devices, with a low standard error of measurement (%FM <1%, FM 0.54−0.87 kg, and FFM 0.58−0.84 kg) [40].

For longitudinal assessments, it is important to recognize that any predicted changes in body composition must exceed both the relative technical error of the technique/device and the biological variability within individuals to be considered statistically significant at the group mean level. The MDC, therefore, represents the smallest change a technique/device can precisely detect, determined by multiple factors as outlined below. For example, if the calculated MDC is 1 kg, then in a 70 kg man with 50 kg of FFM, these devices may only detect a change in FFM if it increases or decreases by 1 kg.

The validity of SF- and MF-BIA measurements depends on several factors, including the specific device and software used, the characteristics of the population being assessed, the characteristics of the prediction equation development sample, the evaluation protocol and measurement conditions, and the reference method [41]. Most BIA prediction equations are less accurate when applied to populations other than those for which they were developed [42,43]. Nevertheless, some BIA devices incorporate equations that have been validated against the reference standard 4-component model in diverse samples varying by race/ethnicity [36]. Software updates that refine prediction equations may alter body composition estimates; therefore, the same software version should be used for cross-sectional and longitudinal analyses, particularly when access to raw data is restricted. When this is not feasible and raw data are unavailable, in vivo cross-calibration with correction factors is recommended. Regarding evaluation protocols and reference methods, one study reported that MF-BIA equations achieved clinically acceptable accuracy for assessing whole-body SMM compared with MRI, but their accuracy was considerably lower for smaller regions, such as the arms [27]. In cross-validation studies, it is essential to use measurements from the same BIA device and software and the same reference body composition method for which the prediction equations were originally developed to ensure the validity of the results. Furthermore, most commercial BIA devices use undisclosed, proprietary equations to estimate body composition, often referred to as a “black box” approach, and significant differences in body composition estimation have been observed across different devices [39,44]. This holds true for raw impedance and PhA measures and their use in published prediction equations, as additional error is introduced when different technologies or configurations are used across bioimpedance devices [26].

Studies have shown that segmental SF-BIA and MF-BIA or BIS improved body composition prediction compared with whole-body SF-BIA [27,[45], [46], [47]]. For example, using the 8-electrode method with one particular MF-BIA device that allows for segmental analysis of arms and legs was found to outperform whole-body SMM estimation compared with SF-BIA measurements based on wrist-ankle placement with 4 electrodes in healthy adults [27]. Moreover, FFM estimated from an equation developed for an 8-electrode, segmental MF-BIA device was comparable in terms of validity and precision with other 2-component reference methods such as ADP or deuterium dilution in a healthy population sample [36]. Nevertheless, caution is needed in interpreting these findings, as segmental bioimpedance may compound errors in body composition estimates depending on the measurement approach and equations used.

Other factors influencing both the reliability and validity of bioimpedance methods include changes in body fluid distribution caused by acute or chronic disease, acute water intake [48], exercise, food ingestion [21], accumulation of excess extracellular fluids (e.g., edema or ascites), and circadian variations [49]. Fluid shifts caused by postural changes, such as transitioning from upright to supine, affect both segmental and whole-body impedance measurements, primarily reflecting changes in leg impedance and total body FFM [50]. Fluid shifts occur gradually after postural changes (change from orthostatic to supine), with ≥5 min required for TBW stabilization and longer durations needed for ECW to equilibrate [51]. In the presence of disease, fluid shifts may require additional time to stabilize, potentially extending the period needed before accurate bioimpedance measurements can be obtained [52]. Importantly, although postural changes may influence bioimpedance results, no differences in the goodness-of-fit of SF-BIA and MF-BIA equations between measurements in the standing and supine positions were observed when subjects lay down for 10 min before supine measurements [27,39]. On the basis of these findings, ambulatory individuals undergoing supine bioimpedance should lay down for ∼5–10 min, and assessors should record the time between assuming the supine position and measurement to ensure standardization at follow-up [12]. Studies have also evaluated the validity of SF-BIA and MF-BIA for estimating body fluid compartments against established dilution methods in healthy adults. Both SF-BIA and MF-BIA demonstrated agreement with deuterium dilution for assessing TBW [36,53,54]. However, only MF-BIA has shown agreement with sodium bromide for ECW [36], as SF-BIA is limited to estimating TBW.

### Bioimpedance-based longitudinal body composition assessment

Bioimpedance methods are well suited for repeated evaluations as measurements are highly reproducible and allow for rapid, convenient, radiation-free, and noninvasive analysis of body composition. The accessibility of bioimpedance methods in terms of low cost and portability, coupled with the speed and relative ease of measurement, makes these methods attractive choices for tracking longitudinal body composition changes. However, caution must be taken to ensure the appropriateness of employing these methods in longitudinal studies as technical measurement error and biological variation need to be taken into account. Body composition changes should be interpreted within the broader clinical context, because substantial differences may reflect violations of assumptions or inconsistencies in measurement procedures and conditions rather than true physiological change. Similarly, when considering large epidemiological or interventional studies, it is important to have a clear assessment and understanding of the degree of interdevice variability if multiple devices or different brands are utilized, as they are often not interchangeable [24,39]. Longitudinal monitoring requires the use of the same device (or a validated equivalent), identical electrode type and placement, the same analysis mode, and ideally the same prediction equation. Switching between modes or equations may introduce artificial changes that do not reflect true physiological variation. Although the use of different devices is not recommended in longitudinal multicenter studies, prevailing circumstances may require their use; in such cases, investigators should provide a comparison of results obtained from each device using a sample of the population under investigation.

As with any repeated measures study, it is important to maintain consistent measurement conditions, such as time of day, body position, ambient temperature, and fasting status to ensure reliability and accuracy. For example, changes in body position influence fluid distribution across compartments, requiring ≥5 min for fluid stabilization before measuring TBW, and even longer for accurate assessment of ICW and ECW [51,55]. In one study, BIS measurements indicated a ∼3% decrease in ECW and a ∼5% increase in ICW after 30 min in a supine position compared with a semiupright posture [55]. Skin temperature also affects measurements: a 6.5°C increase was shown to lower impedance measured by MF-BIA, leading to an overestimation of TBW and a corresponding 13% underestimation of FM% [56]. Although conducted under experimental conditions, and with temperature differences unlikely in healthy individuals, the findings indicate that MF-BIA measurements may be skewed when obtained during fever or hypothermia. Furthermore, one study reported no significant changes in BIA measurements when room temperature was maintained between 20°C and 25°C; outside this range, alterations in skin blood flow appeared to explain the inverse relationship between ambient air temperature and resistance [57]. This temperature range is typical of most clinical settings, whereas humidity appears to have a smaller influence. Postprandial effects have similarly been observed, with impedance values obtained via both SF-BIA and MF-BIA decreasing after food intake, an effect that may persist for 2–4 h postprandially [58]. Attention should also be paid to the interpretation of longitudinal bioimpedance data, particularly in circumstances where the underlying assumptions of the method are compromised, such as in situations where hydration status is likely to change. Such a change may occur when moving between a healthy and pathological state (e.g., progression to, or treatment of, critical illness), during an intervention (e.g., weight loss or gain), or as the result of a normal physiological process (e.g., aging). These situations are described in more detail under “Special considerations for bioimpedance in specific populations/conditions.”

### Standards of bioimpedance

#### Body composition assessment

**FIGURE 3:**
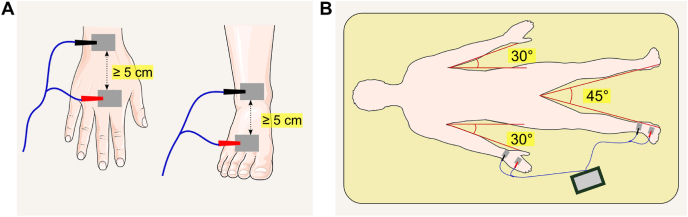
Proper electrode placement and body positioning for bioimpedance assessment. (A) Electrodes with a surface area of ≥4 cm^2^ should be positioned ≥5 cm apart. If this spacing is not feasible, distal electrodes may be placed ≥3 cm away from proximal electrodes, which must remain in their standard anatomical locations. For longitudinal assessments, it is essential to measure and document the electrode distances to ensure consistency in repeated measurements. (B) When conducting assessments in the supine position, the arms should be positioned ≥30° away from the trunk, and the legs should be separated by 45°. Wider angles may be necessary to ensure separation in certain populations. Images retrieved from smart.servier.com.

Fluctuations in fluid levels, even within a single day, underscore the importance of consistent measurement timing to improve accuracy and reliability. Although the side of the body used for measurement typically has minimal impact, SF-BIA assessments are conventionally performed on the right side (Figure 3). For consistency in serial assessments, it is recommended to use the same measurement sites over time. Exceptions include individuals with regional edema, limb amputation, or significant muscular asymmetry such as those who have suffered a stroke with subsequent unilateral muscle atrophy or athletes with dominant-side hypertrophy, where follow-up measurements should be taken on the same side initially used to ensure comparability. Further recommendations are provided elsewhere [12].

#### Data presentation

A summary of bioimpedance assessment reporting recommendations is provided in Box 2. When reporting studies that assess body composition using BIA or BIS, it is essential to include detailed methodological information to ensure reproducibility and accurate interpretation. Authors should report participant characteristics, the standardized premeasurement protocol, and details about the bioimpedance equipment and electrodes used. Authors should also specify whether the measurement was whole-body or segmental, and the subject’s position during assessment (supine or standing, arms not touching the body, and feet position). Reporting and standardizing the time of day and, where possible, ambient conditions, such as room temperature and humidity, is also encouraged to support consistency in assessments. Reporting of hydration status is also recommended; however, although urine specific gravity can be used to assess hydration (values <1.020 indicating euhydration), its clinical utility is limited because it requires midstream collection of a first-morning urine sample, which is not always feasible or available [59]. Furthermore, it is important to state the equation used to estimate body components (if available) and quality control procedures. Adjustments for individual variability, such as expressing mass values relative to body weight or as powers of height (e.g., height^2^) should also be specified. Finally, although not listed as essential in Box 2, it is recommended that studies report repeatability or precision data from replicate measurements in a subset of participants. When reported, precision should be expressed as %CV based on ≥3 repeated measurements performed on the same individual, under consistent conditions. Same-day measurements primarily reflect measurement precision (i.e., intraday variability), whereas measurements obtained on different days (i.e., interday variability) are more informative for estimating the MDC required to identify true longitudinal change. In BIA research, intraday reliability is commonly reported; however, interday reliability and MDC are rarely reported.

BOX 2 **Essential reporting elements for publications of studies assessing body composition by bioimpedance methods**

• Participant characteristics, including sex, age, race/ethnicity, health status, and likelihood of experiencing edema or ascites.
• Standardized premeasurement protocol must be clearly described, including factors such as fasting status, hydration, recent physical activity, medication use, caffeinated drinks/stimulants use, and the duration of rest in a supine or standing position before measurement to allow for fluid equilibrium.
• Bioimpedance equipment model/version, manufacturer’s name, and software used (if available).
• Frequency used, whole-body or segmental, and subject position (supine vs. standing).
• Electrode type, placement, and number (e.g., 4, 8).
• Environmental conditions and the time of day at which the assessment was conducted.
• Prediction equation used to estimate body composition (if available), whether population-specific correction indices were applied (e.g., standard hydration index), and the reference standard and population against which the equation was validated.
• Body composition estimates and raw values (if available).
• Whether any quality control test was performed, the frequency of such checks, and confirmation that the device passed before measurements were taken (failure to pass quality control test should preclude use of the device for measurement).

Alt-text: BOX 2

### Comparing findings across bioimpedance devices

As noted earlier, results from different bioimpedance device brands or models are not interchangeable [25], and therefore, findings should not be compared without proper cross-validation of prediction equations. Although a 50 kHz frequency is often included in MF-BIA and BIS devices, this may not be the case in all devices, limiting the comparison of raw impedance measures across systems [26].

### Selecting an appropriate bioimpedance device

When selecting a bioimpedance device for clinical or research use, it is critical to understand the technical specifications that underpin measurement accuracy and applicability. SF-BIA, MF-BIA, and BIS each differ in their capabilities for estimating body composition and fluid compartments. Additional technical features to consider include whether the system provides whole-body or segmental measurements, the type of electrodes used (e.g., adhesive compared with fixed/stable contact), and the frequencies measured. Practical constraints such as subject weight limits, age, posture requirements (i.e., standing compared with supine), and overall equipment footprint (portable compared with fixed/stationary systems) may influence the choice depending on the target population and intended setting.

Equally important is the nature and format of the device’s outputs, including whether raw data are provided (Box 3) and whether results are available digitally, through printed reports, or integrated with other software. The number of decimal places displayed does not reflect a device’s accuracy or precision, nor does it indicate superiority over other devices. In most real-world settings, the measurement error typically exceeds the reported resolution, limiting the practical significance of additional decimal places. The clinical or research relevance of the reported parameters (e.g., ECW, FFM) should be carefully evaluated. Decision-makers should critically evaluate whether the device uses proprietary prediction equations or allows customization with published, population-specific models. Caution should be taken when some manufacturers market their bioimpedance devices as directly measuring body composition in product brochures, user manuals, and conference presentations. This is scientifically inaccurate and a more appropriate terminology would be “estimate,” as these devices estimate body composition using prediction equations or modeling approaches. In addition, validation studies commonly report correlation coefficients to demonstrate agreement with reference methods. Although these correlations are often strong at the population level, correlation alone does not support accuracy assessment; appropriate evaluation of agreement requires methods such as Bland-Altman analysis, which are not consistently reported, potentially limiting a full assessment of device accuracy at the individual level, usually necessary in clinical practice [12,60,61]. Furthermore, claims that bioimpedance devices are more accurate because of the use of very high frequencies (e.g., beyond ∼1000 kHz) are not supported, as additional information on tissue conductivity tends to plateau at higher frequencies, whereas the risk of measurement artifacts and model instability may increase. Ultimately, selection should be guided by a balance of technical capabilities, usability, and evidence-based validity to meet the specific needs of the user [62].

BOX 3 **Best practices for ensuring transparency and data access in bioimpedance devices**

• A major concern in the application of bioimpedance methods is the limited transparency regarding analysis of raw impedance data. Many commercial devices restrict access to raw measurements (e.g., resistance and reactance) or provide them only in premium models at additional cost. This practice is inappropriate, as it hinders clinicians and researchers from independently applying validated prediction equations, verifying methodological accuracy, or tailoring analyses for specific populations.
• Access to and reporting of raw data are essential for ensuring scientific rigor, reproducibility, and appropriate interpretation of results in both clinical and research settings. This level of access should be provided without additional cost.
• Claims by some bioimpedance device manufacturers of directly measuring body composition are not scientifically accurate, as body composition is estimated using either prediction equations (i.e., it is indirectly measured) or biophysical modeling. In addition, these manufacturers lack transparency regarding device measurement configurations and validation methods, which restricts scientific evaluation and prevents users from fully understanding how body composition estimates are generated, thereby limiting informed decision-making.

Alt-text: BOX 3

### Special considerations for bioimpedance in specific populations/conditions

Because of the portability of most devices, bioimpedance methods can be applied across a wide range of clinical settings, including hospitals, outpatient facilities, and field environments. However, there are important considerations and potential contraindications when using bioimpedance in certain scenarios, as outlined below. It is also important to note that all bioimpedance-derived estimates are subject to 5 types of errors, as summarized by Piccoli, 2014: “the impedance measurement error, the regression error, the intrinsic error of the reference method, the electric-volume model error, and the biological variability among subjects” [63]. In this section, we focus primarily on the possible technical errors encountered in adults with specific conditions.

#### Obesity and rapid weight changes

Individuals with obesity often exhibit altered body shape because of excess adiposity, which may accumulate predominantly in the abdominal region, or around the hips and thighs. The trunk accounts for ∼10% of total body impedance but contributes nearly 50% of the body’s conductive mass, highlighting a key limitation of bioimpedance methods in accurately capturing abdominal composition [64]. This discrepancy stems from the fact that the human body cannot be considered a uniform cylinder composed of a homogenous material, an underlying assumption of bioimpedance (Figure 2A) [21]. Altered body shape, particularly in individuals with excess adiposity, also interferes with the uniform passage of electrical current without shortcuts through defined body segments, such as arms, legs, and trunk. To direct current flow through the intended pathways during supine measurements, insulating materials, such as a towel or rolled cloth or foam pad, may need to be placed between the thighs or under the arms of an individual in supine position; this would allow electrical currents to flow through the desired routes. However, using such materials in a standing position is more challenging, which may compromise measurement accuracy. Moreover, individuals with higher %FM tend to have greater absolute total body and ECW volumes, and their FFM contains ∼76% water compared with the standard 73.2% [[65], [66], [67]]. Issues such as those noted above have resulted in overestimation of FM and a corresponding underestimation of FFM when TBW-based estimates are used to assess body composition [64]. These inaccuracies are reflected in the wide limits of agreement observed between bioimpedance body composition estimates and those obtained from reference methods [[68], [69], [70], [71], [72]]. Adjusting algorithms based on BMI or waist circumference or using equations specifically developed for individuals with obesity may help improve accuracy [67,69].

To date, most studies using SF- or MF-BIA to monitor weight loss have been performed in individuals with obesity undergoing weight-loss interventions or bariatric surgery. Studies utilizing reference methods show that rapid weight loss after a dietary intervention or bariatric surgery decreases FFM, FM, and TBW, whereas its impact on ECW and ICW varies [65,73]. One study comparing changes in body composition measured by SF-BIA compared with a 3-component model reported the advantage of BIA over BMI for predicting changes in %FM [65]. Notably, the equation by Lukaski et al. [74] and the proprietary BIA equation used in the device showed the strongest agreement with the reference method, whereas the Segal et al. [75] equation, despite being developed for individuals with severe obesity, did not show comparable accuracy [65]. However, estimates of FM, FFM, or TBW derived from various SF-BIA and MF-BIA equations showed low bias but wide limits of agreement when compared with reference methods, regardless of whether weight loss was achieved through gastric bypass [76] or dietary intervention [73,77]. These inaccuracies are linked to the same limitations previously discussed in individuals with obesity. Consequently, the use of SF- or MF-BIA for tracking (longitudinal) body composition changes in individuals with obesity undergoing weight loss remains problematic. These methods tend to demonstrate stronger correlations in cross-sectional studies than in longitudinal applications [78]. Notably, underestimation of FM at baseline (before weight or fat loss) can lead to underestimation of FM loss during weight-loss interventions, particularly in individuals with severe obesity [79]. Furthermore, BIA methods cannot detect changes in muscle composition [80], which may negatively impact functionality, or in VAT [81], which is closely associated with metabolic disease risk.

Few studies have evaluated the accuracy of BIA methods in the context of weight gain. In individuals with anorexia nervosa, changes in FM measured by tetrapolar SF-BIA did not significantly correlate with skinfold thickness measurements [82]. Similarly, in individuals with obesity who regained weight after prior weight loss, SF-BIA demonstrated wide limits of agreement when compared with a 4-component model [77]. Although SF-BIA and MF-BIA may not accurately capture changes in body composition during periods of rapid weight fluctuation, they can still serve as useful motivational tools during follow-up, if measurements are consistently performed using the same device and prediction equation. Notably, the European Society for Clinical Nutrition and Metabolism (ESPEN) guidelines support the use of SF-BIA for monitoring body composition [64], particularly when changes in body components are expected to exceed their respective MDC threshold [12].

The accuracy of BIS for tracking changes in fluid compartments has been studied in the context of rapid weight loss after bariatric surgery [83,84]. At the group level, changes estimated by BIS were comparable with those measured by deuterium dilution for TBW and ICW calculated based on sodium bromide estimates of ECW after 6 wk [83]. However, individual-level variability in ICW estimates was high. In another longitudinal study, BIS-derived ECW changes at 2 wk, 3 mo, and 1 y after surgery did not differ from those measured by bromide dilution, whereas BIS significantly differed from deuterium dilution in assessing TBW changes [84].

#### Hydration status and edema

The assumption of stable hydration status inherent to SF-BIA is clearly violated in conditions where ascites or edema are likely to appear or resolve (e.g., individuals becoming critically ill or those with cirrhosis). In these cases, the body deviates from the assumption of a homogenous conductive cylinder because of excess fluid accumulation in specific compartments. This altered distribution of extracellular and intracellular fluid compromises the accuracy of body composition estimates, particularly FFM, by SF-BIA. For example, significant fluid removal through paracentesis in individuals with cirrhosis could not be detected by SF-BIA [85], raising concerns for studies aiming to track body composition over time.

MF-BIA and BIS theoretically offer a more comprehensive assessment of TBW, particularly in the differentiation between ICW and ECW compartments. Although BIS has demonstrated better agreement with reference methods [86], MF-BIA equations can underestimate TBW in individuals undergoing hemodialysis [87]. Still, some MF-BIA devices have demonstrated good reproducibility in detecting acute fluid shifts, such as changes after hemodialysis [88] or transitions from overhydration to normal fluid balance (euvolemia) [89]. However, its consistency is poor when measuring transitions from dehydration to euvolemia and does not fully overcome the limitations associated with overhydration [90]; thus, tracking dynamic fluid changes remains difficult. Moreover, the variability in excess TBW because of disease progression or interventions such as paracentesis, dialysis, or diuretics further complicates hydration assessment. These limitations pose important challenges for (longitudinal) body composition monitoring in both research and clinical settings, including the intensive care unit (ICU) [91]. Nevertheless, raw bioimpedance measurements can remain informative in these conditions.

The validity of BIS for assessing fluid compartments has been investigated under varying hydration conditions. One study assessed the accuracy of BIS for detecting changes in ECW in comparison to bromide dilution during a 4-d fluid restriction and rehydration protocol in healthy young adults [92]. No significant differences were observed between the two methods for estimating ECW under conditions of dehydration and rehydration. These findings are consistent with results from a study exploring varying hydration states, which reported good agreement between BIS and deuterium dilution for TBW, as well as between BIS and bromide dilution for ECW [93]. Although strong coefficients of determination were observed across overhydrated, euhydrated, and dehydrated conditions, wide confidence intervals suggest limitations in the accuracy of BIS at the individual level, a limitation that has been consistently reported when bioimpedance measures are compared with reference methods in individuals with altered adiposity or hydration status and not specific to BIS. In contrast, evidence regarding the accuracy of BIS in patients undergoing dialysis remains inconsistent [94,95].

#### Pregnancy

MF-BIA and BIS techniques are among the most frequently used methods to measure body composition during pregnancy [96,97], although these methods cannot differentiate between maternal and fetal tissues. In early pregnancy, women typically experience modest gains in both FM and FFM as measured by reference method [98]. As pregnancy progresses into the third trimester, weight gain accelerates, with increases in FFM, particularly protein mass and ECW, concentrated in the trunk. FFM hydration also increases, whereas changes in FM are more variable. The weight gain of the fetal unit (placenta, amniotic fluid, and fetus) parallels maternal weight gain.

Compared with isotopic dilution methods, BIS has been shown to accurately estimate TBW during early pregnancy up to week 14, but it underestimates TBW and ECW at week 32 [97]. Similarly, when compared with ADP, MF-BIA tends to underestimate FM in the first trimester and overestimate it in the third trimester [99]. These errors may be the result of the limited ability to accurately quantify small changes in trunk FFM composition because of the minimal contribution to whole-body impedance, as noted previously. Similarly, to previously discussed, it is important to recognize that the body cannot be assumed to be a uniform cylinder composed of homogeneous material, particularly in the later stages of pregnancy, when significant changes in body shape and fluid distribution occur.

#### Other conditions

The use of bioimpedance in individuals with metallic implants remains a somewhat contentious topic, with limited and inconclusive evidence available. Although some manufacturers caution against measurements in those with implants because of potential interference (e.g., increased conductance resulting in lower body impedance), others report minimal impact, particularly with smaller implants such as dental fillings or orthopedic plates [100,101]. Metallic implants may limit the validity of bioimpedance assessments when they substantially alter the electrical current pathway; their presence should be documented, and results interpreted with caution. However, the accuracy of bioimpedance methods in individuals with larger or more centrally located metallic implants has not been fully explored. Given these uncertainties, caution is advised when interpreting results in such populations, and further research is needed to clarify the extent of any measurement bias. Although evidence is limited, silicone breast implants have been shown to affect SF-BIA body composition estimates, particularly by increasing resistance and FM [102].

Safety considerations for bioimpedance assessments in individuals with implanted cardiac electronic devices (e.g., pacemakers and implantable cardioverter defibrillators) remain an area of ongoing evaluation. Although guidelines and device manufacturers recommend monitoring cardiac function in these individuals [64], recent evidence suggests that bioimpedance devices are generally safe and do not induce acute or long-term electromagnetic interference [[103], [104], [105], [106]]. This safety profile has also been demonstrated in patients with end-stage heart failure, without clinically relevant arrhythmias observed within 48 h after BIA assessment [107]. However, in the US, to date, FDA has not cleared any bioimpedance devices for use in patients with pacemakers or implantable cardiac devices.

## Dual-Energy X-Ray Absorptiometry

DXA is an imaging modality that estimates body composition using a 3-component model. Clinical indications for the use of DXA in body composition assessment [108] and a summary of the positions of the International Society for Clinical Densitometry [109] are provided elsewhere.

Box 4 outlines key considerations for DXA-based body composition assessment. Table 2 summarizes the technical and practical characteristics of this method, whereas best-use scenarios and common pitfalls are presented in Supplemental Boxes 5 and 6, respectively. Practices to avoid are summarized in Supplemental Box 7, and key research gaps in the field are outlined in Supplemental Box 8.

BOX 4 **Key points on body composition assessment using DXA**

• Well-established clinical and research method for assessing whole-body and regional body composition.
• Involves very low radiation exposure and supports repeated assessments, although scan frequency should be guided by clinical/research needs and follow the principle of minimizing unnecessary exposure.
• High precision but accuracy varies with the reference method being used, the evaluated population, and the DXA equipment (manufacturer and model).
• Scan positioning, mode, calibration, and software version strongly influence results and require longitudinal consistency.
• Intersystem differences require attention; when equipment or software varies, in vivo cross-calibration is recommended and must be reported if not performed.
• Hemiscans can estimate whole-body composition when size limits are exceeded but should not be used if meaningful right-left asymmetry is present.
• Fluid changes impact lean soft tissue and fat-free mass but only minimally influence estimates of fat mass and bone mineral content.

Abbreviation: DXA, dual-energy X-ray absorptiometry.

Alt-text: BOX 4

**TABLE 2:** Summary of technical and practical characteristics of body composition assessment using dual-energy X-ray absorptiometry.

Body components	Precision/accuracy	Key factors affecting precision/accuracy	Practical considerations	Contraindications
FM, FFM, LST, BMC	High precision High accuracy	System: software, scan mode, calibration Operator: training, scan analysis Subject: movement, body size, adiposity/trunk thickness, hydration and fluid changes (impact LST, FFM), excess breast tissue, breast implant	Stationary Subject size limitations: weight (∼114–283.5 kg), height (∼1.98 m), width (∼0.67 m) Low radiation exposure (<0.01 mSv) ∼10–20 for whole-body scans, with preparation time High device cost; moderate per-test cost	Pregnancy Individuals exceeding the system’s weight limits, as specified by the manufacturer Individuals with implants, casts, or other nonhuman artifacts in scan area Use in certain populations is subject to local regulations

Abbreviations: BMC, bone mineral content; FFM, fat-free mass; FM, fat mass; LST, lean soft tissue.

### Principles and terminology of DXA

**FIGURE 4:**
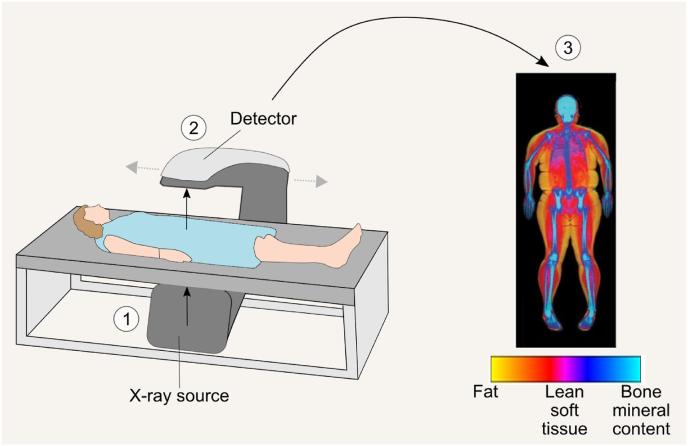
Body composition assessment using dual-energy X-ray absorptiometry (DXA). DXA systems emit X-rays at 2 distinct energy levels, which pass through the body and are detected by a photon detector. This detector measures the amount of energy absorbed by different body components. On the basis of these measurements, individual pixels are classified into fat mass, lean soft tissue, and bone mineral content.

Although DEXA is a commonly used variant of DXA, past consensus groups have encouraged the use of DXA because it follows the format of DXA’s historical predecessor technology, dual-photon absorptiometry [110]. DXA systems generate X-rays at 2 photon energy peaks that are attenuated uniquely by a material’s atomic number and density as they pass through the body [111,112] (Figure 4). Each picture element, or pixel, can be assigned to 1 of 3 components [FM; lean soft tissue (LST); BMC] based on their unique X-ray attenuation characteristics. The component FFM is calculated as the sum of BMC and LST or as the difference between body weight and FM, as explained in our previous publication [15]. These 3 components are reported either for the total body or subregions, such as the arms, legs, trunk, and head.

FM measured in the abdominal region, often referred to as the android region, can be further divided into VAT and subcutaneous AT (SAT). Modern DXA systems place a region of interest (ROI) in the abdominal region and sets demarcations on the abdominal wall to separate VAT and SAT to provide an estimate of VAT [113]. Note that the terms VAT and SAT are commonly used because DXA-derived estimates are calibrated against CT- and MRI-defined AT compartments [114,115]. Although DXA does not directly measure anatomical fat depots, it uses FM-based signals and proprietary algorithms to estimate VAT and SAT. Therefore, referring to these outputs as DXA-estimated VAT and SAT is appropriate, provided it is clear that they represent modeled anatomical compartments rather than direct anatomical measurements.

Regarding FFM components, DXA reports BMC and LST separately. A frequent question is whether LST should instead be termed “lean soft mass.” LST reflects the non-BMC, nonfat soft-tissue compartment defined by DXA based on X-ray attenuation and regional segmentation, rather than a discrete anatomical tissue or a purely molecular mass. The term “tissue” distinguishes this DXA-specific compartment from the broader and method-dependent concept of lean mass. For this reason, the term LST is preferred.

ALST, the sum of the LST in the arms and legs, can be used as a proxy for appendicular SMM or to estimate whole-body skeletal muscle [116]. LST in the extremities, however, is not exclusively skeletal muscle as it also includes lean components from skin, connective tissue, and nonfat components of AT [117]. Appendicular skeletal muscle is part of ALST and can be calculated empirically based on estimates from CT or MRI. Although the term “appendicular skeletal muscle” has been considered to be an acceptable alternative for DXA ALST within certain contexts, it is contingent upon a clear explanation, for instance, in the methods section of a research paper that appendicular skeletal muscle was calculated from DXA ALST using a previously published prediction equation derived from CT or MRI (e.g., DXA-predicted appendicular skeletal muscle from CT). Along with BMC, DXA estimates “areal” bone mineral density (BMD) for whole-body and the appendages, and further subdivides the trunk regions into ribs, thoracic spine, lumbar spine, and pelvis. Areal BMD is distinct from physical density, which is mass per unit volume of a material. DXA does not report volumetric BMD, except for special scan protocols of the spine. We once again refer readers to our previous publications for guidance on appropriate DXA-related terminology [15,117].

### Reliability and validity of DXA

Overall, DXA shows high precision, with CVs ranging from 0.9% to 3% for %FM, 0.7% to 3.4% for FM, 0.6% to 1.6% for BMC, and 0.4% to 2.2% for LST [118,119]. However, reliability and validity vary with the reference method being used, the evaluated population, and the DXA equipment (manufacturer and model) [119]. Thus, it is recommended that inter-rater CVs remain <2% for %FM, 3% for absolute FM, and 2% for LST to ensure measurement reliability [119].

The current reference method for FM is the 4-component body volume model [120]. DXA provides valid estimates when evaluated against 4-component FM with SEs of estimate between 2% and 3% depending on the population [121,122]. Total FM estimates by DXA systems are strongly correlated (*r* = 0.99) with total body AT as measured by MRI [123]. Strong associations are also observed between VAT estimated by DXA compared with CT (coefficient of determination, *R*^2^ = 0.96 [124]; correlation coefficient, *r* = 0.93 [125]) and MRI (*r* = 0.88–0.94) [115,126]. Half-body DXA scans (hemiscans) can provide accurate whole-body estimates [127] when subjects do not fit on the scanner platform; however, hemiscans should not be used for whole-body composition if there is any noticeable imbalance between the right and left sides.

Factors that can influence DXA accuracy and precision, including MDC, encompass technician training, scanning protocols, scan analysis, system software, calibration procedures, scan mode, subject movement during scanning, and subject body size/adiposity level [128]. Small amounts of food or fluid intake before a DXA scan, typically <100–150 g, have minimal effects on FM and FFM estimates and generally fall within the measurement error of the system. In contrast, larger intakes exceeding 500 g can acutely alter body mass, FM, and FFM estimates and may influence clinical interpretation [[129], [130], [131]]. Dietary manipulations such as carbohydrate loading can transiently increase FFM and reduce %FM, supporting the need for standardized or controlled diets before DXA testing [132,133]. Changes in hydration may influence LST (and subsequently FFM) but have little effect on FM and BMC when using consistent protocols [134]. The degree of control required may vary by context; in athletes, small amounts of food intake before scanning may be acceptable, whereas larger dietary manipulations should be avoided [129]. In clinical settings, consistent pretesting dietary and fluid intake restrictions may be needed to prevent bias in longitudinal assessments.

### DXA-based longitudinal body composition assessment

DXA is a well-recognized clinical and research method for measuring longitudinal changes in whole-body and regional FM, LST, and BMC [135]. To be interpreted as true biological change, observed differences must exceed measurement variability. Because absolute precision errors are expressed in kilograms, their proportional impact varies with body size, which in turn affects the estimated CV and the minimum detectable change across populations. For example, root mean square errors of ∼400–600 g for FM and 500–880 g for FFM have been reported, but these values may represent a larger relative error in smaller clinical populations and a smaller relative error in larger athletic populations [[136], [137], [138]]. Accordingly, device precision should be established in a representative sample to determine the population-specific sensitivity of longitudinal assessments.

Examples of practical considerations of the CV have been explored across different body regions. After 2 wk of bed rest, a 5% decrease in leg LST measured by DXA corresponded to a 5% reduction in leg muscle volume by MRI and a 6% reduction in thigh muscle cross-sectional area (CSA) by CT [139]. This finding supports the accuracy of DXA in monitoring changes in leg LST, as the CV was 1.3%, corresponding to a MDC of 3.6%, and thus, the observed change was considered clinically significant. Repeated scans of a whole-body phantom, conducted within a 5-mo interval, showed a coefficient of variability of 3.1% for BMC and 3.9% for BMD [140], representing an MDC of 8.6% and 10.8%, respectively. Changes in VAT after bariatric surgery were underestimated by DXA compared with MRI [141]. Body weight measured on a calibrated scale should be compared with the DXA total mass and the sum of regional masses as part of a quality control procedure. Agreement should be within ±1 kg at each time point.

### Standards

#### Body composition assessment

Calibration should be conducted as recommended by the manufacturer. Phantoms with anthropometric characteristics are preferred for longitudinal assessment of single equipment performance; these phantoms should be scanned daily. Whole-body phantoms scanning should be performed multiple times a week, where available. Acquiring whole-body phantoms that accurately replicate BMC, LST, and FM is challenging, and none currently provide absolute accuracy. These phantoms are often modular by design, incorporating various shapes of white high-density polyethylene to simulate FM, alongside polyvinylchloride to represent LST and aluminum to represent BMC. DXA calibration also often involves the use of stearic acid, a nonessential lipid (triglyceride), which affects the quantification of FM, BMD, and LST and further compounds the complexity of body composition terminology.

DXA operation manuals should be consulted for the weight and height limits of the equipment. At present, weight limits of DXA systems range from upper limits of ∼114–283.5 kg depending on manufacturer [135,142]. Most DXA equipment can scan individuals ≤1.98 m in height and 0.67 m in width [143], although some models accommodate ≤2.28 m in height and 1.37 m in width [135]. As mentioned above, hemiscans can be used in individuals with a large body size who do not fit within the scanner guides [127]. Another alternative is to impute the values from one extremity to the “missing” extremity that does not fit within the scan window. For tall individuals, 2 scans can be performed; one that includes the feet but excludes the head, and the other that includes the head but excludes the feet [143]. The missing head values from the first scan can then be manually imputed using data from the second scan.

Different scan modes (e.g., “high performance” mode) are available on some systems and may differ in body composition estimates; consult manufacturer guidelines when selecting among scan modes to determine the relevant populations and/or benefits of these features. A typical whole-body scan usually takes under 10 min depending on the subject size and DXA equipment. The scan should be conducted with subjects wearing a hospital gown and without any attached jewelry. Loose fitting clothing, without zippers or underwired bra, is also acceptable. Individuals with metal implants will likely have inaccurate whole-body and site-specific DXA scan results because of the interference that the metal can cause with the X-ray beams, although regions without implants will not be affected. Calcium supplements should be discontinued 24 h before the scan. Although acute feeding has had minimal impact on body composition measurements [130], food intake should be limited ≥2 h before the scan for standardization; an overnight fast is ideal for maintaining consistency particularly in the research setting, although it may not be practical in the clinical setting. Individuals who have had any medical imaging procedure that included contrast agents should wait for ≥2 wk before undergoing body composition evaluation by DXA.

DXA scans are typically administered by a technologist or medical imaging professional who has been trained and certified by an accredited institution. It is crucial to consult local regulations to determine the specific qualifications required for operating DXA equipment, as there can be significant regional variation. Some regions may allow for a broader range of trained individuals to conduct scans, whereas others mandate certification or specify that a radiologic technologist or physician must perform the procedure. On the basis of the experience of some of our expert group members, regulatory policies may restrict the populations eligible for DXA assessment. In some regions, DXA is permitted only for specific clinical indications (e.g., osteoporosis), or requires special approval for other groups, such as individuals with obesity or pediatric populations. These requirements can differ greatly among countries and even within regions of the same country, underscoring the need for understanding and adherence to local standards and practices.

Altering the image guidelines, for example in the presence of scoliosis, can have a significant effect on the software calculation of LST, FM and BMC in any given anatomical site, and neighboring sites, and should only be done when absolutely necessary, allowing the software to determine limb, head and trunk divisions. This is particularly relevant in longitudinal studies.

Available data indicate that DXA radiation doses are low and comparable with natural background radiation exposure (<10 μSv) [135]. The radiation dose from a typical DXA examination is very low, comparable with natural background radiation received over 1–2 d. Although the principle of minimizing unnecessary exposure (as low as reasonably achievable, ALARA) always applies, the number of scans should be guided by clinical or research necessity. In many settings, including weight loss programs and longitudinal monitoring, >2 whole-body scans per year may be entirely appropriate. Additional details can be found on the International Atomic Energy Agency website [144]. Women who are pregnant should not undergo DXA examinations; pregnancy screening is recommended for women of childbearing age before proceeding with DXA.

#### Data presentation

A summary of DXA assessment reporting recommendations is provided in Box 5. The DXA system, manufacturer’s name, and software version should be reported along with the facilities’ CV for each body component evaluated. DXA reports should include values for BMI, BMD, BMC, total mass, total LST, total FM, and total %FM [145]. When extracting and interpreting results from the DXA equipment output, care should be taken as terminology in downloadable results can differ depending on the equipment system and software version [15]. For example, some General Electric DXA equipment data output uses the term “Lean” to depict LST. Hologic DXA equipment uses both the terms “Lean mass” and “Lean + BMC” in its results sheet; thus, one would need to manually subtract BMC to obtain LST if the “Lean mass” output is not available or is presented to represent “FFM.” When reporting results, clearly defining the terms of interest and ensuring the use of proper terminology is essential. FM, LST, and BMC can be expressed as a percentage of body mass. Powers of height (e.g., height^2^) are often used to adjust measures of mass for between-individual differences in height.

BOX 5 **Essential reporting elements for publications involving body composition assessment by DXA**

• Participant characteristics, including sex, age, race/ethnicity, and health status.
• Standardized premeasurement protocol must be clearly described, including factors such as fasting duration, discontinuation of calcium supplements, hydration status, and the absence of recent contrast exposure.
• DXA system, manufacturer’s name, and software version.
• Quality control procedures, such as daily phantom calibration and, when applicable, cross-calibration of DXA systems across different sites or software versions.
• Facility-specific coefficient of variation for each body component assessed.
• Scanning protocol (e.g., use of hemiscans or duplicate scans, imputing missing limbs).
• Additional regions assessed should be indicated and described.
• Indicate whether participants with metal implants were excluded, and if included, report the number and type of cases.

Abbreviation: DXA, dual-energy X-ray absorptiometry.

Alt-text: BOX 5

### Comparing findings across DXA devices and/or sites

Body composition results across DXA manufacturers, systems, and software versions may differ and should be considered in multicenter studies in which data are combined for analysis. When DXA equipment and software vary, *in vivo* cross-calibration between study sites is advised for observational studies. Phantom cross-calibration of DXA equipment of different technologies is not currently supported because of insufficient availability of cross-calibration phantoms, and in vivo cross-calibration might not be feasible for sites spread across a wide geographic area [146]. Despite these challenges, research sites are strongly encouraged to perform cross-calibration, whenever possible, to eliminate potential site- or device-specific variability that may confound data interpretation. When cross-calibration is not conducted, this must be clearly stated in publications. In clinical settings, individuals requiring longitudinal assessments should be advised to undergo DXA scans on the same equipment used at their most recent evaluation [146]. Cross-calibration approaches are described elsewhere [146]. Cross-calibration should be performed according to established recommendations when updating to a new system or hardware [109,147,148]. Multiple studies exist that provide device- and system-specific calibrations for whole-body or regional bone or body composition measures [113,147,[149], [150], [151], [152]].

### Selecting an appropriate DXA device

Differences in hardware, software, and calibration methods exist between DXA systems and can affect measurement outputs and intersystem comparability [153]. Key differences between manufacturers include beam technology, table size, and weight capacity. Some systems use narrow-angle fan beam technology and generally offer larger tables with higher weight limits—features that may be better suited for scanning taller individuals or those with obesity. Other common DXA systems, on the other hand, employ wide-angle fan beams and more compact scan tables, which can reduce scan time and enhance image acquisition efficiency in individuals representative of the general population or high-throughput clinical environments.

Within each manufacturer, further variation exists between device models, including differences in pixel density, image resolution, and radiation exposure. Newer models can offer higher-resolution imaging and improved soft-tissue discrimination, beneficial for people with obesity or pediatric populations, but delivering a slightly higher radiation dose. Models from the same manufacturer can also differ in image quality and performance, with some offering higher resolution and shorter scan times. Overall, although BMD and body composition estimates are highly correlated across systems, hardware and software differences must be considered when comparing values across platforms or conducting multicenter trials.

Other factors to consider when selecting DXA equipment include methods for analyzing scans with metal implants, scan time and radiation dose, local regulatory requirements, availability of service contracts and maintenance, access to reference databases, and the system’s physical footprint.

### Special considerations for DXA in specific populations/conditions

#### Obesity and rapid weight changes

When assessing individuals with obesity, it is essential to ensure that their body weight falls within the scanner’s weight limit and that they fit entirely within the scanning field to ensure accurate measurements (see below for an exception). Suitability assessments should be conducted with care and sensitivity to avoid contributing to weight-related stigma. Positioning their hands in a mid-prone position can aid in fitting within the scan frame and in achieving proper shoulder alignment. Excess breast tissue is also a concern in individuals with severe obesity, as it may lead to overestimation of LST in the arms; consistent positioning in serial assessments and careful interpretation are recommended. For those who exceed the dimensions of the scanning field but are within the scanner’s weight limit, hemiscans can be employed as an alternative, provided special considerations, mentioned above, are taken into account. Additionally, it is recommended to select the scan mode based on trunk thickness, as specified by the equipment manufacturer, because automatic mode may not choose the appropriate setting.

Another aspect to consider involves individuals who are experiencing rapid weight loss because of illness or pharmaceutical or surgical treatments for managing obesity. Although DXA is effective in tracking changes over time for most body components, with potential caveats for VAT, changes in body composition must exceed the equipment measurement error (defined by MDC) to be deemed clinically significant, despite meeting statistical thresholds. To minimize measurement variability introduced by changes in tissue thickness or X-ray attenuation (e.g., beam hardening), it is essential to maintain consistent scan settings (including scan mode) and positioning protocols across timepoints, even after substantial weight loss.

#### Hydration status and edema

Edema, ascites, and other accumulated bodily fluids are common in clinical populations and will be estimated by DXA as part of the LST (and subsequently FFM) component. Fluid changes will therefore impact LST and FFM but only minimally influence estimates of FM and BMC [129,134]. Exercise-induced alterations in hydration can impact both body mass and LST estimations and should be carefully controlled [134]. As noted earlier, DXA evaluations are not recommended in pregnancy.

#### Management of individuals with embedded objects

DXA is not recommended for evaluating body composition in people with implants, casts, or other nonhuman objects that might interfere with X-ray attenuation. The presence of implants can affect the precision of the body composition measurements, especially if the implants are located within the scan regions of interest for body composition assessment [154]. Hemiscans or software-specific imputation analyses are advised for those who have had hip or knee replacements [155]. Evidence does, however, indicate that imputation analysis, which substitutes metal artifacts with the unaffected side (if available), affects bone measures; nevertheless, the impact on LST and FM are controversial [156,157]. Similarly, breast implants may influence whole-body bone measures, with the trunk area being affected the most, even though changes in trunk LST and FM have been reported to be within measurement errors [158].

## Computerized Tomography

CT is widely regarded as a reference method for body composition assessment because of its high spatial resolution, which ensures accuracy, and its ability to capture detailed anatomical information. CT also offers the advantage of opportunistic analysis using existing clinical scans, provides a broad range of measurable features, and enables visualization of distinct body composition phenotypes, making it a valuable and versatile body composition assessment method. Nevertheless, CT-based body composition analysis has been mostly limited to clinical populations, particularly in oncology, where existing scans can be repurposed without exposing individuals to additional radiation or cost. In this context, CT images can be used to assess low muscle mass and support the diagnosis and management of malnutrition, sarcopenia, and cachexia, as well as to predict health outcomes [[7], [8], [9], [10], [11],[159], [160], [161], [162]]. Longitudinal monitoring is also constrained by the availability of routine clinical imaging, as additional scans solely for body composition would result in unnecessary radiation exposure and increased clinical/financial burden.

Box 6 outlines key considerations for CT-based body composition assessment. Table 3 summarizes the technical and practical characteristics of this method, whereas best-use scenarios and common pitfalls are presented in Supplemental Boxes 9 and 10, respectively. Practices to avoid are summarized in Supplemental Box 11, and key research gaps in the field are outlined in Supplemental Box 12.

BOX 6 **Key points on body composition assessment using computerized tomography (CT)**

• Useful in clinical populations with existing scans in medical records, enabling diagnosis and longitudinal monitoring of body composition abnormalities with detailed tissue differentiation.
• Multiple commercial and open-source tools support manual to fully automated analysis.
• Measurement accuracy depends more on consistent slice selection than operator experience. Analyze ≥2 slices when detection of small changes is critical.
• Unenhanced scans acquired in the same contrast phase scans are preferred; contrast-enhanced scans should be used cautiously and reserved for segmentation, when appropriate.
• Consistent equipment and protocols (e.g., contrast phase, tube voltage) should be used for cross-sectional and longitudinal analyses. Cross-calibration is recommended when scanners or acquisition protocols differ.
• Intermuscular adipose tissue is the preferred terminology to describe all adipose tissue located between muscles.

Alt-text: BOX 6

**TABLE 3:** Summary of technical and practical characteristics of body composition assessment using computerized tomography.

Body components	Precision/accuracy	Key factors affecting precision/accuracy	Practical considerations	Contraindications
Adipose tissue, skeletal muscle	High precision High accuracy	System: image quality, contrast enhancement, phase, tube voltage, slice thickness Operator: image selection and analysis Subject: movement, fluid overload, cardiac leads, metal artifacts, positioning between follow-up scans, measurement site	Stationary Subject size limitations: weight (∼200–300 kg), diameter (∼60–85 cm) High radiation exposure (1–10 mSv) ∼15 min for image analysis with semiautomated software; ∼<5 min for image analysis with fully automated High device cost; moderate per-test cost	Pregnancy Individuals exceeding the system size limits Not suitable for body composition assessment alone

### Principles and terminology of CT

**FIGURE 5:**
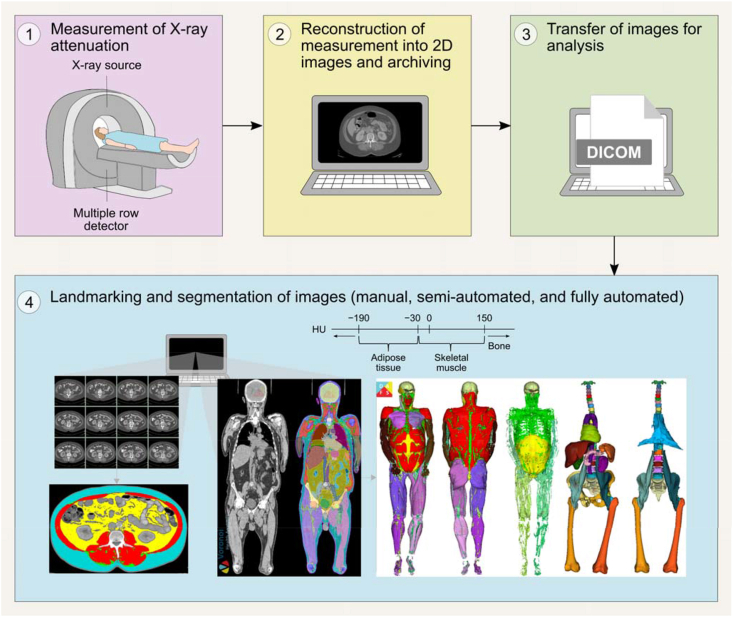
Typical workflow of body composition assessment using computerized tomography (CT), with each colored box representing a distinct step in the process. The workflow begins with a CT scan performed in a clinical or research setting [1], capturing X-ray attenuation data from body tissues. These data are reconstructed into 2D images, which are typically archived for future analysis. When body composition assessment is required, images are transferred [typically in Digital Imaging and Communications in Medicine (DICOM) format] to specialized software for image selection (i.e., landmarking) and tissue segmentation [3, 4]. During segmentation, pixels are classified as adipose tissue or skeletal muscle based on predefined Hounsfield unit (HU) ranges or anatomical boundaries. The number of pixels corresponding to each tissue type within a defined region of interest is summed, and the cross-sectional area is determined by multiplying this pixel count by the pixel surface area. Landmarking and segmentation can be conducted manually, semiautomatically, or fully automatically, depending on the software used [164]. The fully automated segmentation image shown in the bottom right corner is a selected example; adapted with permission from Voronoi Health Analytics Inc, Canada.

CT is an imaging method that uses X-ray technology to create detailed cross-sectional images of the body. Body components are differentiated at the tissue-organ level based on their radiodensity measured in Hounsfield Units (HU), where tissues with higher radiodensity appear lighter (e.g., skeletal muscle, bone), and those with lower radiodensity (e.g., AT) appear darker on cross-sectional images [163]. Although the term “density” is often used in the context of CT-based body composition analysis, it should be avoided, as HU does not represent physical density in terms of mass or volume. HU radiodensity refers to photons in the X-ray, and the density of photons hitting the detectors determines the light and dark nature of the images. Thus, HU is a normalized unitless scalar that reflects the ratio of linear attenuation coefficients, which are measured in units of 1/distance, and more accurately represent “radiodensity,” the material’s ability to attenuate X-rays. The process of CT image acquisition and analysis is detailed in Figure 5.

CT measures tissue-organ level body composition; as such, the correct terms are “adipose tissue” (as opposed to “fat mass”), and “skeletal muscle” (as opposed to “lean mass,” “fat-free mass,” or “lean soft tissue”). The only exception is when prediction equations are used to estimate other components, such as FFM or LST. We again refer the reader to our previous publication for a detailed discussion on recommended terminology [15]. Many organs and tissues can also be quantified, including bone mineral radiodensity, liver, spleen, kidney, pancreas, and among others [165]; however, these are not the focus of the current paper.

Although many factors can influence tissue radiodensity, tissue composition is particularly significant. AT, primarily composed of fat (i.e., triglycerides), exhibits a lower radiodensity than fat-free skeletal muscle, which is composed mainly of water, electrolytes, and protein; this terminology has been discussed in detail elsewhere [15]. Under normal conditions adipocytes are found in small clusters and as individual cells between muscles fascicles and muscle groups, collectively referred to as intermuscular AT (IMAT) [166]. In contrast, intramuscular AT (intraMAT) refers to adipocytes located within muscles but outside muscle fibers, accounting for the extramyocellular lipid (EMCL) detected and visualized using magnetic resonance spectroscopy (MRS). However, intraMAT is not readily visible on CT. Fat is also present in small amounts and in the form of intracellular lipid droplets within muscle fibers. These droplets are enclosed by a phospholipid bilayer and contain a triglyceride-rich core, forming what is known as intramyocellular lipid (IMCL), which can also be quantified by MRS. These definitions differ slightly from that used in our previous manuscript in the series [15], reflecting ongoing refinement and incremental gains in conceptual clarity as discussions in the field evolve. Nevertheless, the terms IMAT and intraMAT are still frequently used interchangeably in the literature, highlighting persistent ambiguity in their application. Therefore, future research and consensus efforts are needed to clearly define and distinguish between these 2 components.

**FIGURE 6:**
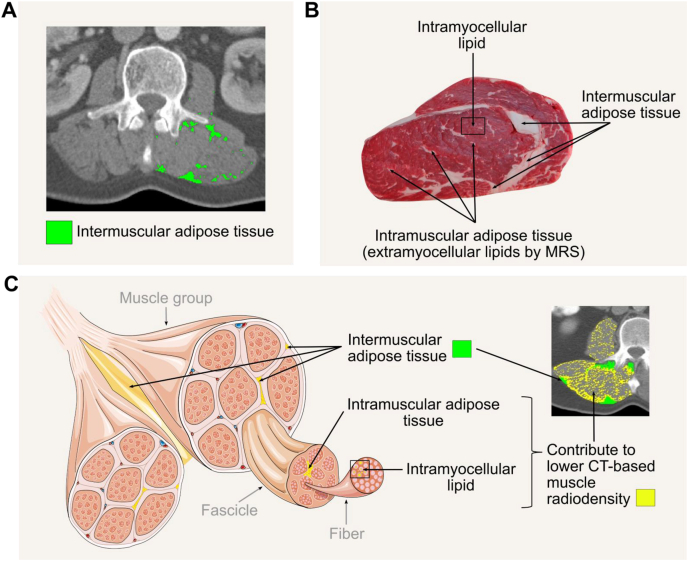
Visual analogy of intermuscular adipose tissue (IMAT), comparing computerized tomography (CT) imaging of human muscle (A) with marbling in a beef steak (B) and a schematic of skeletal muscle structure highlighting the anatomical locations of different adipose depots (C). IMAT refers to clusters and individual adipocytes located between muscle fascicles and muscle groups. Intramuscular adipose tissue (intraMAT) represents adipocytes within muscles but outside muscle fibers, detected as extramyocellular lipids (EMCL) by magnetic resonance spectroscopy (MRS). Intramyocellular lipids (IMCL) are intracellular triglyceride droplets within muscle fibers. Although IMCL contributes to reduced muscle radiodensity, it cannot be distinguished from IMAT on CT. Therefore, IMAT is recommended as a broader term encompassing all adipose tissue, including individual adipocytes, found in muscles. Images retrieved from smart.servier.com.

Excessive fat accumulation within skeletal muscle, reflecting the relative expansion of IMCL and IMAT triglycerides, is termed myosteatosis. This condition presents as reduced muscle radiodensity on CT, which ultimately represents muscle composition. CT-based muscle radiodensity therefore reflects fat infiltration from both intraMAT and IMCL. However, because of typical CT spatial resolutions of ∼0.1−0.5 mm, it cannot distinguish between muscle pixels with high compared with low IMCL content [167]. Thus, although IMCL contributes to muscle radiodensity, it cannot be differentiated from IMAT in CT-based analysis (Figure 6).

We suggest the term “muscle composition” to be used instead of “muscle quality” when referring to muscle radiodensity. Although “muscle composition” can generally refer to muscle fiber types and morphological phenotypes, in the context of CT-based analysis, it specifically refers to myosteatosis. Furthermore, muscle quality is an evolving and broader concept that encompasses multiple aspects of skeletal muscle, such as its composition, morphology, architecture, and function [[168], [169], [170]]. AT radiodensity has been recently reported in the medical literature [160,171]. Lower radiodensity in this context is often indicative of adipocyte hypertrophy and an elevated fat content within cells [160,172]. Conversely, higher AT radiodensity relates to lower fat content in adipocytes.

Despite similar nomenclature, peripheral quantitative computed tomography (pQCT) differs from CT and is not the focus of this manuscript. Briefly, pQCT was originally developed to assess volumetric BMD and content, but it also generates single cross-sectional images that enable measurement of skeletal muscle CSA, muscle radiodensity, and AT in peripheral limbs [173]. These measurements are obtained using threshold-based segmentation to identify tissue borders, followed by postprocessing required to derive area and density measures [173]. Although evidence remains limited, pQCT appears to be precise, with reported root mean square CV of 2.1%–3.7% for muscle CSA and 0.7%–1.9% for muscle radiodensity [174]. pQCT has also demonstrated accuracy for detecting longitudinal changes in mid-thigh muscle CSA compared with MRI, with no significant differences between methods after a 6-wk resistance training program [175]. Compared with CT, pQCT offers greater portability, lower equipment and per-scan costs, and substantially lower radiation exposure (<0.0015 mSv per scan compared with 1–10 mSv of CT) [173]. Future work from our group may focus on standardizing acquisition and analysis protocols for pQCT-based body composition assessment.

### Reliability and validity

CT is an accurate body composition method that agrees with actual volume and mass of cadaver organs with a CV of ± 3%−5% [176]. This accuracy and precision also apply with regard to measures of appendicular skeletal muscle and SAT, with ∼2% reproducibility for the latter [177]. Good agreement has also been found between cadaver and CT-assessed abdominal AT, with a correlation coefficient of 0.89 for SAT and 0.88 for VAT [178]. For thigh skeletal muscle CSA, CT showed a percent error of 0.5% at the distal third and 4.9% at the mid-third compared with photographic assessment of a single cadaver [179]. However, another study of 3 cadavers found that CT overestimated thigh skeletal muscle CSA by 10%–20% relative to anatomical assessment, likely due to subjective interpretations of boundaries between adjacent muscle bellies [180]. Unlike other body composition methods, CT reliability assessments typically include only the image selection and analysis procedures, without incorporating repeated image acquisition (i.e., re-scanning); thus, our understanding of the reproducibility of CT is somewhat limited. CT mass and volume analyses are highly reproducible based on repeat image selection and analysis, with SEs of estimate of 0.96%−0.99% for SAT and 0.67−0.99 for VAT [163]. Measurement error for skeletal muscle CSA at the third lumbar vertebrae (L3) ranged from 1.3% to 2.0% [[181], [182], [183], [184], [185], [186]]. Inter-rater CV ranged from 0.6% to 2.4% for skeletal muscle CSA at L3 [181,[187], [188], [189], [190]].

Several factors can influence the validity and precision of CT-based body composition assessments. When using CT scans opportunistically from patient records, low-quality images are frequently encountered because of movement, fluid overload, cardiac leads, or metal artifacts [191]. Additionally, the X-ray beam passing through the ulna, radius, or humerus bones can impact image quality. These factors can cause streak artifacts that result in missing data and may affect assessments, particularly tissue radiodensity. We recommend excluding low-quality images from analysis to improve assessment integrity.

Regarding precision, one study found that most variability between repeated measures of skeletal muscle arose from 2 evaluators selecting different slices at the same site because of anatomic variations [192], underscoring the need for greater standardization, training, and expertise in anatomical landmarks for manual slice selection. Although training is necessary, experience segmenting the images does not appear to influence assessment as much as selecting the slice, as novice observers were as reliable as more experienced observers when segmenting images [193]. Another variability source was the manual adjustment of segmentation after automated thresholding. Additionally, for longitudinal assessments, changes in subject positioning between scans, especially when treatment influences posture, can lead to inconsistencies, as the exact same slice may not be captured in repeated scans. To reduce variability, the authors recommend using the same evaluator for all image analyses, clearly defining criteria for selecting axial slices with discussions in cases of abnormal anatomy, and analyzing 2 slices in studies where detecting small effects is critical [192].

### CT-based longitudinal body composition assessment

CT imaging is a valuable method for monitoring body composition changes over time in individuals with cancer and other chronic diseases, as scans obtained for treatment evaluation and disease monitoring are often readily available in medical records and allow for detailed tissue differentiation [161]. To accurately track these changes and determine their significance, appropriate precision metrics must be established. A previous study reported the MDC for skeletal muscle CSA, measured twice in a single slice at different sites, at baseline and follow-up (7–8 mo), as follows: 1.8 cm^2^ in upper arm, 0.9 cm^2^ in thigh, 2.1 cm^2^ at fourth thoracic vertebrae (T4), and 2.6 cm^2^ at L3 [194]. For AT, the MDC was generally higher: 4.1 cm^2^ in upper arm, 13.1 cm^2^ in thigh, 18.0 cm^2^ at T4, and 3.8 cm^2^ at L3 [194]. The precision of changes in body composition based on serial CT scans remains to be established [159], as repeat scans are rarely performed within timeframes short enough to assume biological stability, and using the same equipment and operator. Because precision measures are influenced by evaluator, software, slice selection, and other factors, each setting should establish its own MDC values for image analysis, ideally using repeated imaging acquisition when possible. Site selection is a major potential source of measurement error; therefore, this step should be carefully standardized. Each measurement site and segmentation approach (e.g., manual compared with automated) requires a predefined and reproducible method for slice selection, particularly in single slice and longitudinal protocols. For example, when manually selecting an L3 slice, the assessor should scroll through the CT series from the lungs to identify the first lumbar vertebrae (L1), continue caudally to L3, and select the mid-vertebral L3 slice using consistent anatomical landmarks (i.e., the slice with the thickest transverse processes and bilateral symmetry). Scrolling should continue to confirm the positions of L4 and L5. In longitudinal assessments, the current scan should be directly compared with the prior image, especially bony landmarks, to ensure that measurements are obtained at the same anatomical level across time. Similar to MRI clinical trials, site selection may also be guided by CT calibration phantom scans to confirm scanner performance across multiple study sites, with routine phantom-based quality assurance maintained throughout longitudinal studies.

The use of standardized CT image acquisition protocols and the choice of measurement site are additional factors that contribute to accurate longitudinal body composition assessment. As discussed later in this section, factors such as contrast enhancement and tube voltage can affect body composition estimates by CT, particularly the radiodensity of tissues. Additionally, single CSA may not depict specific regional or whole-body changes that may occur in health and disease. For example, age-related muscle changes are more pronounced in the lower limbs than in the upper extremities [163,195]. Similarly, although one study in males with head and neck cancer found the thigh to be the most sensitive site for detecting skeletal muscle change, systemic alterations were also observed in other regions including the upper arm, chest, and abdomen, highlighting the potential for broader shifts in body composition beyond a single anatomical site [194].

### Comparing findings across CT scanners and/or protocols

Cross-calibration is recommended when different CT scanners or acquisition protocols are used for body composition assessment. This can be performed using commercially available quality control phantoms or custom phantoms based on published designs [[196], [197], [198]]. Although cross-calibration using *in vivo* data is ideal, it is not feasible to scan individuals solely for quality control purposes because of radiation exposure. Alternatively, a phantom with known HU values can be scanned concurrently with an individual during a study and serve as an internal reference [[199], [200], [201]]. Applying appropriate correction factors after calibration ensures the comparability and validity of measurements across scanners and protocols. After CT software updates, scanner recalibration and verification using a calibration phantom are also strongly recommended and should be performed by a medical physicist to ensure optimal performance.

### Standards of CT

#### Body composition assessment

Selected axial cross-sectional images correlate well with whole-body composition and can be used for body composition assessment [202,203]. As explained elsewhere [204,205], the preferred landmark of interest has traditionally been the L3. In a study by Shen et al. [202], the objective was to find the single image location most accurately reflecting whole-body muscle and AT volumes, and to propose equations to estimate these components. Their methodology involved analyzing images at the L4/L5 (lumbar vertebrae) level, and at positions 5 and 10 cm above, and 5 and 10 cm below this level. The strongest correlation for whole-body skeletal muscle was discovered 5 cm above the L4/L5, approximately corresponding to the L3 area. Although the strongest correlation between a single slice and whole-body AT was found 5 cm below L4/L5 [202], the strongest correlations between VAT area and volume was found at 5–10 cm above L4/L5 in one study [203], [6] and 6 cm above this intervertebral space in another [206]. The use of single-slice areas at mid-L3 was further validated in individuals with colorectal cancer [207]. The CSA of skeletal muscle, VAT, SAT, and IMAT strongly correlated with multislice abdominal volumes and demonstrated similar relationships between area and volume with all-cause mortality [207].

Although studies have evaluated one muscle group (e.g., psoas muscle) at the L3 level instead of total muscle CSA, this approach is not recommended. A single muscle group is not representative of all muscle, which could result in greater error [[208], [209], [210]]. This is corroborated by studies showing no associations of a single muscle group with clinical outcomes, an underestimation of the occurrence of myosteatosis, or a discordance between low muscle mass diagnosed by a single muscle group and total muscle CSA [210,211].

Beyond the commonly used L3 site, thoracic CT scans have also been suggested as a useful landmark for body composition assessment, particularly when abdominal images are not available from medical records [212,213]. Moreover, regions like the cervical area have also been used for this type of analysis [214]. In research settings, the thigh muscle area is utilized to assess longitudinal changes, offering a reliable measure for tracking progression over time. The thigh muscle area is often obtained with pQCT [173].

A variety of commercial and open-source software is available for manual, semiautomated, or fully automated image analysis based on artificial intelligence-based algorithms [159,164,204,215,216]. Variations between software may exist; however, they are unlikely to significantly impact the diagnosis of low muscle mass [193]. Fully automated software can operate in any standard desktop or laptop, providing a multitissue, multiorgan, 3-dimensional segmentation [164]. Every axial cross-sectional slice from a CT image series is segmented and annotated according to its vertebral level, allowing for precise quantification of tissues and organs. The development of such software, which allows for large-scale analyses to be conducted in a fraction of the time required for manual segmentation, marks a significant breakthrough and a new era in CT body composition assessment. We direct the reader to additional sources listing software and studies focused on the investigation of automated and semiautomated software tools for CT scan-based body composition analysis [159,204,217,218]. This is an evolving and dynamic field, and our expert group is aware of numerous leading hospitals and research institutions actively developing their own, in-house software solutions to meet clinical and research demands.

**FIGURE 7:**
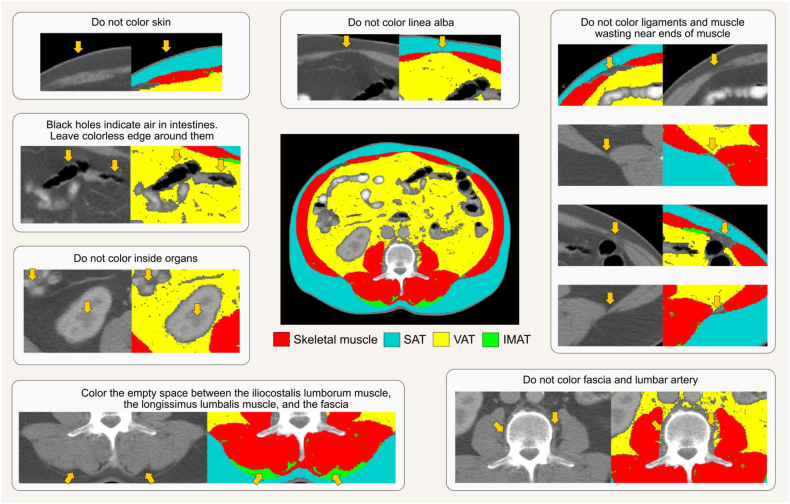
Important considerations for body composition assessment at the third lumbar vertebrae using a computerized tomography (CT) scan. IMAT, intermuscular adipose tissue; SAT, subcutaneous adipose tissue; VAT, visceral adipose tissue.

Algorithms have produced comparable results with those of manual segmentation [159,216], but semiautomated and fully automated segmentation requires a quality control procedure in place to avoid selecting and segmenting poor-quality images, which would otherwise be excluded in manual segmentation. Additional manual corrections of segmentation may also be required during quality control to avoid overestimating tissue areas. For example, intracolonic contents can have HU similar to VAT, potentially leading to mistaken identification as VAT [219]. In large-scale studies, manual corrections for skinfolds (pannus) or SAT cutoffs may not be necessary [159]. Automated segmentation has demonstrated high mean Jaccard scores compared with manual segmentation for images with such issues, indicating strong agreement between automated and reference segmentations [159]. However, scores were moderate for individuals with conditions such as hernia, fluid accumulation, or diastasis recti, representing potential limitations of automated methods in such cases and requiring review of images to maintain data integrity [159]. Manual checks on a subset of images are recommended in population-based studies (Figure 7).

Image acquisition protocols may impact body composition measurements [218,220,221]. This is especially important for measuring muscle radiodensity as well as VAT CSA and radiodensity [222,223]. In many cases, the absorption of the contrast agents, used to highlight physiological deviations, into tissues will increase radiodensity [220,[222], [223], [224]]. This increase in radiodensity may alter how pixels are “viewed” by the imaging software, falsely suggesting better muscle or poorer AT composition; this error in analysis may also contribute to false categorization of individuals with low compared with high muscle radiodensity [225]. For instance, analyzing images from the arterial and venous phase, as opposed to unenhanced CT images, led to significant increases in skeletal muscle area (0.8%–1.7%), skeletal muscle index (SMI, 0.8%–1.8%), and muscle radiodensity (14.8%–21.6%) [226]. Among the diverse contrast agents available, the use of intravenous contrast during the portal venous phase is recommended to be applied for the analysis of images for body composition [220,221,223,225,227].

The impact of contrast is stronger when scans are taken at varying tube voltages, which are adjusted based on subject size to either reduce radiation dose or improve image quality. For scans using the same tube voltage, the difference in skeletal muscle CSA between contrast-enhanced and nonenhanced images was 2.3 ± 1.7 cm^2^; however, when scans used different tube voltages, this difference increased to 7.0 ± 7.5 cm^2^ [221]. Another study observed variations in radiodensity of muscle and AT, SMI, muscle CSA, and the area of muscle with a low radiodensity (−29 to 30 HU) between images acquired with intravenous contrast enhancement at 2 different tube voltages (80 and 140 kV) [228]. Similar acquisition protocols, including the use of contrast agent and tube voltage, are therefore required to compare CT-based body composition results between individuals in cross-sectional analysis, or within individuals in longitudinal analysis [221]. For analyzing tissue radiodensity, it is recommended to use only unenhanced CT images or contrast-enhanced scans acquired in the same phase, whereas the use of enhanced images should be reserved as a reference for tissue segmentation [226].

The influence of slice thickness on image analysis appears minor compared with the factors above. For slices 2–10 mm thick, the maximal mean differences were 1.9% for SMI, 3.3% for the CSA of muscle with low radiodensity (−29 to 30 HU), and 1.5% for AT index, with no variation in muscle radiodensity [220]. Another study found 5 mm slices showed a 1.1% higher muscle CSA but an 11.6% lower muscle radiodensity compared with 2 mm slices [229]. The clinical relevance of these differences remains uncertain and warrants further investigation.

#### Data presentation

A scoping review addressed methodological inconsistencies in skeletal muscle and AT assessments in oncology research [181]. The authors proposed methodology and data reporting standards for longitudinal CT-based body composition analysis. These include specifying inclusion criteria of CT acquisition time points relative to disease trajectories and reporting the MDC. Body composition results should include the actual interval between scans, baseline CSAs (in cm^2^ and cm^2^/m^2^), and changes stratified by sex using absolute and relative metrics. Our group supports these standards and suggests additional recommendations summarized in Box 7.

BOX 7 **Essential reporting elements for publications involving body composition assessment by CT.**

• Participant characteristics, including sex, age, race/ethnicity, and health status.
• CT image acquisition details (e.g., contrast enhanced, noncontrast, positron emission tomography), tube voltage, and slice thickness. Specify whether a contrast agent was used and if so, identify the phase of contrast administration, as this can affect radiodensity and cross-sectional area measurements.
• Analyses of tissue radiodensity should be performed using unenhanced CT images. If contrast-enhanced images are used, all scans should be acquired in the same contrast phase and should be used primarily for tissue segmentation rather than radiodensity evaluation.
• Timing at which CT images were obtained relative to the disease trajectory (e.g., at diagnosis, before treatment initiation, or after treatment completion), and the interval between scans in longitudinal assessments.
• Anatomical location chosen for body composition analysis.
• Name and version of the CT image segmentation software used, and state whether segmented was performed manually, semi-automatic, or automatically.
• Hounsfield unit ranges for tissue segmentation, if applicable.
• Number of evaluators involved in image segmentation, and report intra-observer and interobserver (when applicable) coefficients of variation for each body component. In longitudinal studies, also report the minimal detectable change for image selection and segmentation.
• For automated segmentation, describe any quality control procedures, such as manual pre-screening or post hoc screening of image subsets used to detect any inaccuracies and exclude poor-quality images.
• Application of any manipulation techniques (e.g., estimations, prediction equations) to address incomplete images beyond the field of view.
• A representative segmented image to illustrate how the image was performed.
• Use intermuscular adipose tissue (IMAT) as the preferred term to describe all adipose tissue located in muscles, visible with CT.

Abbreviation: CT, computerized tomography.

Alt-text: BOX 7

### Selecting an appropriate software for CT-based body composition analysis

A variety of open-source and commercial software options are available for manual, semiautomated, or fully automated CT-based body composition segmentation, and this landscape continues to evolve with ongoing updates and new developments. When selecting among these tools, we recommend evaluating factors such as precision, cost-effectiveness, required training, and the time required to complete assessments.

### Special considerations for CT in specific populations/conditions

CT imaging has become increasingly popular in clinical settings for its accuracy, reliability, and the ability to access high-resolution images obtained for diagnostic or monitoring purposes in clinical populations, such as cancer, chronic obstructive pulmonary disease, cirrhosis, kidney diseases, and trauma/ICU [159,161,181,185,188,205,212,230,231]. Although image quality challenges may arise in these individuals, these limitations do not preclude CT-based body composition assessment and can often be addressed through careful image review and standardized analysis and reporting procedures.

#### Obesity and rapid weight changes

Although CT is not routinely indicated for body composition assessment in obesity management, it can be used opportunistically in oncology or other obesity-associated conditions. In such cases, careful consideration of method-specific limitations in this population and strategies to minimize their impact are essential. CT scanners have weight (∼200 to ∼300 kg) and bore size (∼60–85 cm) limits, and individuals exceeding these limits cannot be accommodated [232]. Another challenge in acquiring CT images in individuals with excess body weight is the image noise and artifacts caused by beam hardening and photon starvation, which result from the subject’s body pressing against the scanner’s top and sides [233]. Image quality issues can also occur because the increased thickness of soft tissue prolongs the time it takes for photons to pass through the body, leading to motion artifacts [232] that may make differentiating muscle and AT more challenging. Excess body weight may also impact subject positioning, consequently limiting the field of view and leading to incomplete body composition assessment. Additionally, when subjects exceed the field of view, radiologists may crop the images to focus specifically on the internal organs [232]. One approach to maximize the use of routinely acquired CT images in individuals with obesity is to apply imputation approaches when a portion of the image is missing, such as duplicating values from the complete side, particularly for skeletal muscle and SAT.

#### Hydration status and edema

Abdominal edema is common in individuals with cancer, kidney disease, liver disease, and in those who are critically ill. Fluid retention impacts CT body composition assessment because of alterations in tissue radiodensity. As such, extra fluid can lower HU values of affected lean tissue, causing it to appear less dense. Although changes in muscle radiodensity were not significantly correlated with changes in edema classification in one study [231], edema may lower muscle radiodensity, leading to misinterpretation as increased fat infiltration and poorer muscle composition, while also overestimating SMI and muscle CSA [231,234]. For instance, in critically ill patients with abdominal sepsis, increased edema formation was significantly associated with an increase in SMI [231]. In another study in individuals with cirrhosis, positive associations between skeletal muscle CSA and FFM hydration, independent of total body protein, were also found [234]. Fluid retention can also impact SAT assessment, as edema often spreads throughout AT, making its boundaries unclear [235]. Therefore, it is important to document and report clinical signs of fluid retention and recent fluid shifts at the time of imaging, and to interpret lower muscle radiodensity with caution when edema is present. In longitudinal studies, comparing scans acquired under similar hydration states, when feasible, can help ensure meaningful interpretation.

## Ultrasound

US is increasingly used for body composition assessment because of its low cost, portability for point-of-care application (particularly in immobile patients, such as the critically ill), and absence of ionizing radiation. These characteristics make it well suited for prospective, longitudinal assessments in healthy and clinical populations. Box 8 outlines key considerations for US-based body composition assessment. Table 4 summarizes the technical and practical characteristics of this method, whereas best-use scenarios and common pitfalls are presented in Supplemental Boxes 13 and 14, respectively. Practices to avoid are summarized in Supplemental Box 15, and key research gaps in the field are outlined in Supplemental Box 16.

BOX 8 **Key points on body composition assessment using ultrasound (US)**

• Low-cost, portable, and free of ionizing radiation, making it ideal for point-of-care and longitudinal body composition assessment in healthy and clinical populations, including critically ill or immobile patients.
• Provides direct and accurate measurements of tissue thickness and cross-sectional area.
• Prediction equations can convert tissue thickness measures into estimates of regional or whole-body tissue volume or mass.
• Well suited for monitoring change in muscle and adipose tissue; careful selection of anatomically and clinically meaningful landmarks is essential for valid longitudinal assessment.
• Muscle echo intensity is a surrogate metric reflecting noncontractile tissue infiltration and is associated with functional and metabolic outcomes.
• Brightness-mode is preferred over amplitude-mode due to its more comprehensive 2-dimensional assessment.
• Anterior landmarks are commonly used in clinical settings because they are more accessible in the supine position at the bedside.

Alt-text: BOX 8

**TABLE 4:** Summary of technical and practical characteristics of body composition assessment using ultrasound.

Body components	Precision/accuracy	Key factors affecting precision/accuracy	Practical considerations	Contraindications
Adipose tissue, skeletal muscle	Low to moderate precision High accuracy	System: configurations, imaging protocols, transducer orientation and angle, image quality, software enhancements Operator: training, landmarking, image acquisition and analysis, transducer pressure and angle Prediction equation (if used): reference standard, population, and compliance with the imaging and analysis protocol Subject: adiposity, measurement site, fluid accumulation	Portable Limited to superficial muscles (3–5 cm field of view) and AT thickness No radiation exposure (0 mSv) ∼15 min for measurement, depending on operator skills and number of measurement sites Low to moderate device cost; low per-test cost	None, but altered hydration or high adiposity may reduce accuracy

### Principles and terminology of US

Brightness-mode (B-mode) US imaging utilizes soundwaves to generate 2-dimensional (2D) cross-sectional images of subcutaneous structures within the body. Although “ultrasonography” refers to the application of US for imaging purposes, “ultrasound” is the preferred term and has been used extensively in clinical practice and scientific literature. Images are generated using a pulse-echo approach, which involves the transmission of high-frequency soundwaves from piezoelectric crystals located in the transducer into the subcutaneous tissues (the pulse) and detection of the reflected soundwaves from underlying structures along its path (the reflected echo) [236]. As the US pulse travels deeper into the underlying tissue and encounters different structures, the soundwave will be reflected to the transducer as an echo at varying signal amplitudes. Echo reflections occur at tissue interfaces, where the reflected signal amplitude is dependent on the change in acoustic impedance (a similar concept to tissue density) between the tissues [236]. These detected US echoes are processed and displayed as 2D images, with more reflective structures appearing hyperechoic (i.e., brighter). Hence, tissue interfaces with large differences in acoustic impedance (e.g., muscle-to-bone or skin-to-AT) will produce high amplitude reflected echoes, which appear as bright structures in the image. Within US images, all structures are displayed using pixels, ranging from 0 (black) to 255 (white). Although US scan time is relatively short, the total image acquisition time typically spans ≤15 min [12] but can vary depending on the operator’s level of expertise, the number of anatomical sites assessed, the use of single compared with repeated measurements, and subject-specific factors.

**FIGURE 8:**
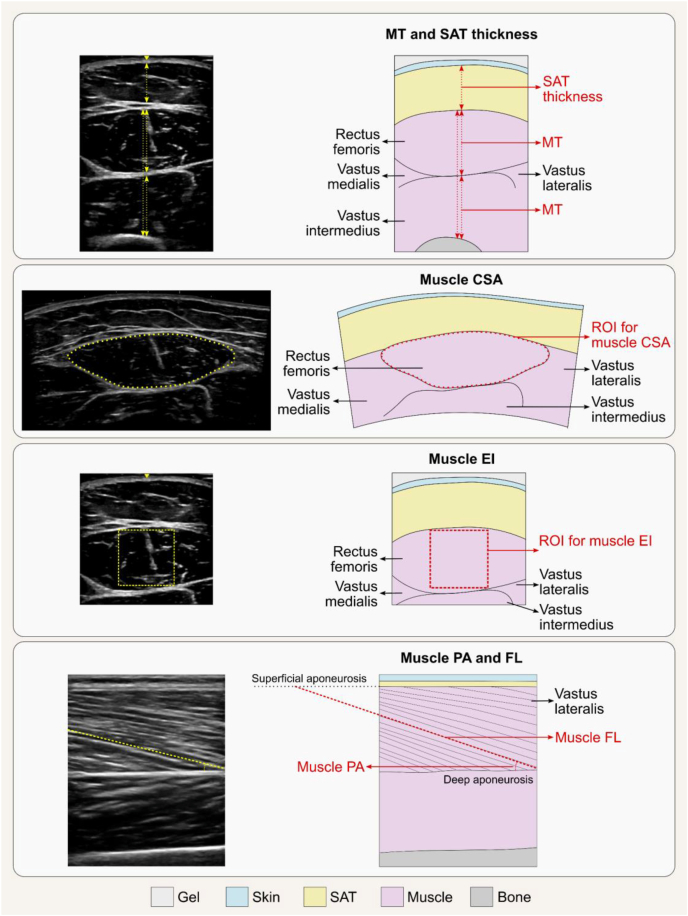
Schematic representation of quantitative tissue assessment and qualitative muscle assessment in a selected body site (mid-thigh) using ultrasound. CSA, cross-sectional area; EI, echo intensity; FL, fascicle length; MT, muscle thickness; PA, pennation angle; ROI, region of interest; SAT, subcutaneous adipose tissue.

2D contrast images can be analyzed either directly on the US device, using built-in analysis software, or by exporting them to an external image processing software. The hyperechoic (i.e., bright) nature of the skin, muscle fascia, and bone is used to differentiate muscle and AT at the tissue-organ level of body composition (Figure 8). The specific metrics that can be extracted for analysis of tissue mass vary but generally include muscle thickness (MT), SAT thickness, and the CSA of single muscles at specific anatomical landmarks [[237], [238], [239], [240], [241], [242]] (Table 5). US is also widely used to evaluate the macroarchitectural properties of muscle, including fascicle length (FL) and pennation angle (PA) [243,244]. Using computer-aided analysis, US can be used to derive surrogate metrics of muscle tissue composition (e.g., infiltration of noncontractile tissues, such as adipose and fibrotic tissues) [239,245,246]. Although SAT thickness is most commonly evaluated, US can also evaluate deep and superficial abdominal SAT, intra-abdominal AT thickness, abdominal wall AT index, preperitoneal AT thickness, mesenteric AT thickness, epicardial AT thickness, and perirenal/nephric AT thickness [247,248]. Although the term “fat” is commonly used to describe these metrics, it is more accurate to refer them as “adipose tissue,” given that US assesses body composition at the tissue-organ level. As mentioned previously, readers are encouraged to consult our guidelines for the appropriate use of body composition terminology [15]. Emerging US technologies are being developed and applied to assess the composition of muscle and AT, such as shear wave elastography (Supplemental Information 2) [249] and speed of sound [250] analysis, but they will not be the focus of this review.

**TABLE 5:** Definitions of common ultrasound-derived metrics for body composition assessment.

Ultrasound metric	Definition
MT	Perpendicular distance (in cm) from the superior to the inferior boundaries of the muscle fascia of a single muscle or muscle group measured in the sagittal or transverse plane at a specific anatomical site.
Muscle CSA	Area (in cm^2^) of a single muscle measured in the transverse plane at a specific anatomical site. This represents the “anatomical” CSA, not the “physiological” CSA, which is more related with force production. Thus, muscle CSA includes the total surface area of the muscle belly, encompassing internal components such as fibrotic and fatty tissues.
SAT thickness	Perpendicular distance (in cm) between the skin-SAT and the SAT-muscle interfaces measured in the sagittal or transverse plane at a specific anatomical site.
Muscle EI	Pixel intensity in a region of interest, reflecting intermuscular adipose and fibrous tissues. Values are reported in arbitrary units, typically ranging from 0 (black) to 255 (white).
PA	The internal angle (in degrees) formed by the insertion of muscle fibers into deep and superficial aponeurosis in a pennated muscle.
FL	Distance (in mm) between the superior and inferior aspects of a single pennated muscle fiber, measured parallel to the lines of collagenous tissue in the sagittal plane. It serves as an indirect estimate of muscle fiber length, except in long muscles composed of serially connected fibers.

Abbreviations: CSA, cross-sectional area; EI, echo intensity; FL, fascicle length; MT, muscle thickness; PA, pennation angle; SAT, subcutaneous adipose tissue.

Depending on the measurement purpose and availability of reference values, prediction equations can be useful for converting raw US metrics to whole-body and regional measures of tissue volume or mass. For example, several prediction equations have been developed using MT to estimate ALST [[251], [252], [253]] or FM from DXA [254], or whole-body and regional skeletal muscle volume based on MRI [255,256]. Thus, although US primarily assesses body composition at the tissue-organ level, prediction equations can extend its estimates to other levels. To promote comparability and reproducibility, we recommend the use of standardized terminology and transparent reporting when referring to US-derived estimates. This includes specifying the anatomical site, imaging protocol, and whether values represent direct measurements (e.g., MT) or derived estimates (e.g., ALST, muscle volume). The terminology used should reflect the estimated compartment.

“Healthy” muscle is generally hypoechoic (i.e., dark), but aging, disuse, and/or disease can lead to infiltration of noncontractile tissues, which alter the appearance of muscle on US images. Infiltration of noncontractile tissues into muscle creates new tissue interfaces (e.g., muscle-AT or muscle-fibrotic tissue) where the US pulse can be reflected as an echo back to the transducer (i.e., be displayed as hyperechoic pixels within the muscle). Although visually present, unlike other imaging modalities at the tissue-organ level (e.g., CT, MRI), US does not have the capacity to differentiate IMAT from contractile tissues. Thus, any metric of muscle size (CSA or MT) will include these noncontractile tissues, limiting their accuracy as a measure of contractile tissue. See our previous work for clarification on AT terminology [15].

Although US cannot directly differentiate between contractile and noncontractile tissues when measuring thickness or area, surrogate metrics [e.g., mean echo intensity (EI)] can provide estimates of noncontractile tissue infiltration [257]. These surrogate metrics typically evaluate the pixels within the muscle fascia, as infiltration of noncontractile tissues will increase the degree of reflection and overall brightness within the muscle borders (Figure 8) [257]. Because the increased brightness cannot be differentiated into specific tissues (e.g., adipose compared with fibrotic), we suggest the term “muscle composition” (instead of “muscle quality”), as it more accurately represents the mixture of tissues within the muscle fascia boundaries and their relevance to biomechanical and physiological functions. Although many computer-based image analysis algorithms are used to assess muscle composition, mean EI is the most widely adopted because of its ease of use with open-source software. This metric is also associated with functional outcomes, such as muscle strength, and metabolic control in healthy and clinical populations [245,258,259]. Additional factors that can influence muscle composition metrics include edema, necrosis, and inflammation. These factors, and their extent, can vary with aging and disease states, potentially affecting muscle composition. However, the specific impact of each factor remains to be fully understood in the context of US imaging.

### Reliability and validity of US

US-derived metrics for muscle and AT quantification are usually considered accurate representations of their respective tissue, as several studies have reported strong positive correlations between tissue thickness or CSA and CT, MRI, and cadaveric dissections [260], even in clinical populations [261]. However, commercially available US devices capture images at a single landmark with a narrow field of view (3–5 cm in width), limiting CSA analysis to small, superficial muscle groups and AT to thickness measurements. Because of these limitations, reference equations incorporating US-derived metrics are often utilized to predict appendicular or whole-body indices of muscle and/or AT mass. These equations incorporate muscle or AT thickness (often in association with anthropometric measurements such as height, body mass, limb length and/or circumferences), in relation to MRI whole-body muscle volume, DXA LST, and CT measures of CSA at the L3.

Although strong associations are generally reported with single landmarks, increasing the number of landmarks evaluated provides stronger associations with appendicular and whole-body derived metrics of muscle and AT mass. Prediction equations have been developed using the MT from ≤9 sites against MRI muscle volume (*R*^2^ = 0.77–0.92) [256], DXA ALST (adjusted *R*^2^ = 0.88–0.97) [251] and DXA LST of the right leg (*R*^2^ = 0.96) [262] in standing postures, as well as 5- and 2-site MT against DXA ALST (*R*^2^ = 0.91; adjusted *R*^2^ = 0.89–0.90, respectively) [252,253] in a supine posture, which is applicable to bedridden individuals. For AT, recent work has demonstrated good to very-good agreement between US-derived measures of %FM and those measured using a 4-component model (*R*^2^ = 0.70), although with an overestimation (∼3.5% higher than 4-component model) [263]. However, the ideal landmark for evaluating muscle and AT masses is not well established and likely differs depending on the body component being evaluated, the population of interest, and the feasibility of the technique for a given population (e.g., ambulatory compared with bedridden). Moreover, although several prediction equations exist for generally healthy adults [251,253,[264], [265], [266]], few are available for clinical populations, limiting the use of US in clinical settings and hindering understanding of how well a single muscle reflects whole-body musculature.

Muscle EI displays moderate correlations with EMCL [267] but not IMCL from MRS, and moderate to strong correlations with IMAT infiltration from MRI [268], suggesting that the reflected echo mainly occurs because of AT infiltration into the muscle fascia rather than lipids per se. However, mean EI cannot be solely considered a measure of AT infiltration, as positive correlations are also observed with fibrotic tissue, muscle necrosis, and inflammation [241,269]. Also, SAT overlaying the muscle is a major confounder of mean EI because of the beam attenuation that occurs in deeper tissues (i.e., pulse scatters and reduces in intensity before reaching the muscle). This beam attenuation factor creates challenges when comparing mean EI across individuals with varying amounts of SAT, even while using the same equipment and settings. Although some authors have developed correction factors for the SAT layer, these are likely specific to the equipment, population, and muscle groups being evaluated.

Although US is considered overall a valid tool for muscle and AT analysis, measurements of reliability are inconsistent. For MT, studies reported that the CV for intrarater analysis ranged from 1.1% to 7.0%, with ICC between 0.81 and 1.00; for inter-rater reliability, the CV ranged from 2.5% to 13.0%, and ICC values between 0.67 and 0.99 [252,[270], [271], [272], [273], [274], [275], [276], [277], [278], [279], [280], [281]]. AT thickness also displays similar intra- and inter-rater reliability, as ICC ranges from 0.7 to 0.97 and CV from 2.0% to 9.0% [270,273,282]. These discrepancies are likely due to the wide variety of assessment protocols and the operator-dependent nature of the method [283]. For instance, novice assessors showed greater variability in SAT thickness measurements compared with experienced assessors, both in inter-rater and intrarater assessments [284].

When evaluating the reliability of an US protocol for body composition analysis, 3 operator-dependent steps are involved: site identification and marking (i.e., landmarking), image acquisition, and image analysis. Image analysis has been most extensively evaluated for reliability, which displays strong intra- and inter-rater reliability in healthy and clinical populations, even among relatively novice raters with minimal training [280]. Often, studies evaluate the reliability of the image acquisition and analysis steps combined, in which multiple images are captured on predefined landmarks, which show generally strong repeatability (ICC >0.8) on test-retesting of several landmarks [285]. However, few studies assess the entire protocol, including re-landmarking, limiting our understanding of its overall repeatability. Recently, Fischer at al. [282] evaluated muscle and AT thickness reliability in inpatients (surgical and medical wards) using a comprehensive protocol that incorporated re-landmarking and evaluated both intrarater and inter-rater reliability across upper arm and thigh sites by evaluators with varying degrees of experience. Despite using the greater trochanter, a more challenging landmark for the anterior thigh than the anterior superior iliac spine, high to very high ICCs (>0.8) were reported for limb-length (i.e., landmarking) measurements, as well as AT and MT assessments. More work is needed to evaluate the day-to-day reliability of the entire protocol across diverse populations.

### US-based longitudinal body composition assessment

US is a practical tool for prospectively monitoring longitudinal changes in body composition, given its accessibility and absence of ionizing radiation. Within clinical settings, it has been primarily used in critical care, where studies have reported muscle atrophy rates of ∼2% per day during the first week of ICU admission [286]. Selecting an appropriate and clinically meaningful landmark is critical for evaluating longitudinal changes. With biological aging, it is generally accepted that lower limb muscle mass is lost at a greater rate than upper limb muscle mass [194]. However, a recent systematic review in patients who were critically ill reported similar rates of atrophy for MT and CSA of the biceps brachii and rectus femoris during the acute phase (i.e., within 2 wk) [286], suggesting a more global muscle degradation process.

Accurately measuring these changes in muscle and AT using US depends on its MDC, which is influenced by multiple factors discussed earlier. Reported MDC for interday MT assessments across select lower limb muscles ranged between 0.01 and 0.09 cm for experienced raters and from 0.07 to 0.19 cm for inexperienced raters [283], underscoring the importance of rater-experience for obtaining valid results. Assuming a quadriceps MT of 3.0 cm in a critically ill patient, a 0.1 cm MDC would represent an ∼3% change from baseline, which is well below the degree of muscle atrophy that would be present within an acute phase in the ICU.

### Standards of US

#### Body composition assessment

A major challenge with US for body composition assessment is the lack of standardized and globally accepted imaging protocols, which hinders cross-study comparisons and the development of normative data for evaluating disease-related changes [287]. Although expert opinion-based guidelines are emerging to standardize assessment protocols for several muscle groups [288,289], differences in clinical settings and patient populations may lead to inconsistencies (e.g., difficulty accessing posterior landmarks in bedridden patients).

**FIGURE 9:**
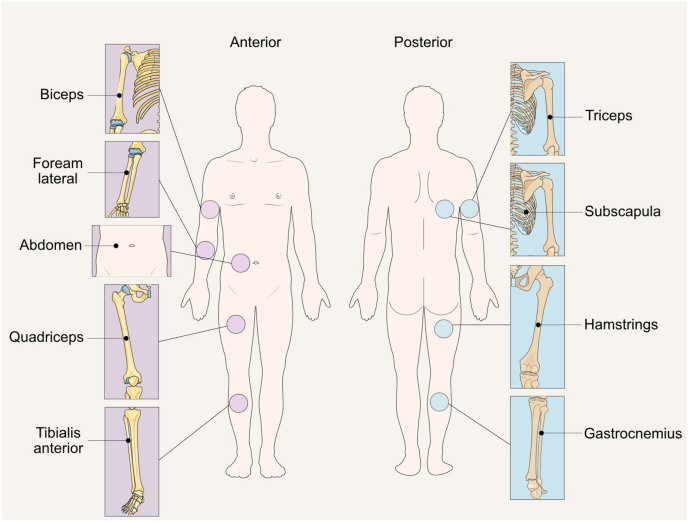
Common measurement sites used in ultrasound-based body composition assessments. Although the classical 9-site protocol includes all of these sites to estimate muscularity [270], some alternative protocols assess only a subset [253,262]. Images retrieved from smart.servier.com.

Assessment time is an important consideration. Although the classical 9-site protocol [270] provides comprehensive and accurate prediction estimates of muscularity, it may be impractical in routine practice (Figure 9). Recent studies indicate that using fewer, more accessible sites does not substantially reduce the accuracy of prediction equations [[251], [252], [253],^266^]. Several of these sites are emerging as preferred options in clinical populations because of their biological relevance, reliability, and feasibility in both standing and supine postures. For instance, anterior landmarks are preferentially used for clinical assessments of skeletal muscle and AT, because of their accessibility in supine posture at the bedside [271,275,[290], [291], [292], [293]]. The anterior and lateral thigh are the most commonly assessed sites for US-based MT and CSA, widely adopted in the critical care literature for their accessibility and sensitivity to atrophy during acute ICU stays [237,260,[294], [295], [296], [297]]. The anterior upper arm, anterior forearm (ulnar MT), and anterior lower leg have also emerged as accessible landmarks, providing strong estimations of muscle mass [296].

Although the adoption of fewer landmarks does not seem to affect reliability or validity, capturing multiple images of each landmark for mean analysis (2–3 per site) can reduce the variability without prohibitively increasing the time required for analysis [251,253,271,293,296,298,299]. Compared with a single measurement, SE of the mean for MT decreased by 25% when using the mean of 2 consecutive measurements, and by 50% when using the mean of 3 measurements [299]. Although evaluators’ experience does affect the reliability of results, standardized training strategies have proven capable of producing sufficiently reliable results [278,280,281,300,301]; however, there are no standardized criteria defining the level of training required to ensure reliable and valid results. Minimal training for non-expert US operators may include instruction on US equipment and probe handling, anatomical structure identification and landmarking of measurement sites, assessment procedures and image analysis, and hands-on practice in individuals with varying body sizes or representative of the target population [302,303]. Building on these components, a comprehensive 20-h training program was developed for critical care physiotherapists to support US-based muscle assessment, yielding ICC values of 0.70−0.76 for inter-rater and intrarater reliability of MT measurements [302].

**FIGURE 10:**
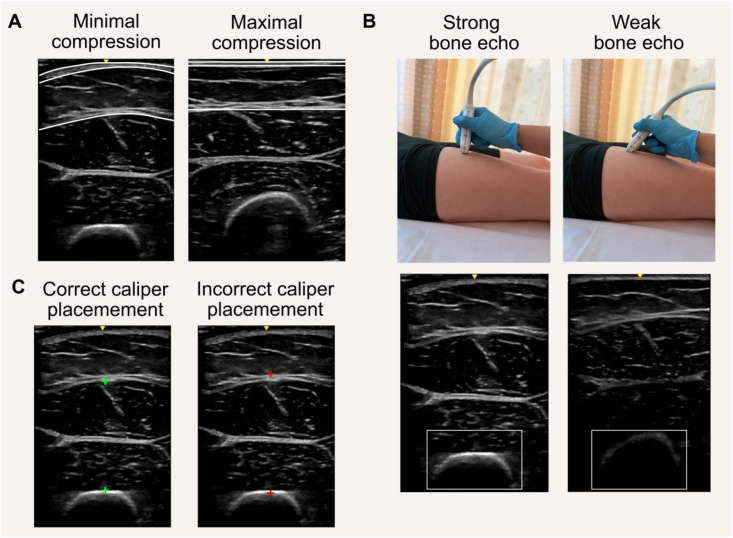
Practical considerations for body composition assessment using ultrasound. (A) A reliable approach to confirm that an ultrasound image was captured with minimal compression is by identifying a visible skin arch in the image. (B) Changes in probe tilt can be identified by the bone echo: correct/neutral probe tilt produces strong bone echo; incorrect tilted/inclined probe produces weak bone echo. (C) Correct placement of calipers within the ultrasound image for accurate measurements of the quadriceps (rectus femoris + vastus intermedius) muscle thickness.

The transducer orientation is another important consideration during image acquisition. Most commonly, the transducer is applied perpendicular to the long axis of the muscle to obtain cross-sectional images of the muscle for assessment of its CSA and thickness. Longitudinal imaging (i.e., parallel to the long axis) provides visualization of the muscle fascicles for analysis of architectural properties (FL and PA—muscle dependent). The pressure applied and probe tilt are additional considerations (Figure 10). We recommend using minimal compression by applying and maintaining a generous amount of conductive gel between the probe and skin surface (see “Hydration Status and Edema” section below for additional discussion), providing concave images of the underlying skin, adipose, and muscle layers (Figure 10) [12,271,304]. A prototype wireless force sensor system has been developed and integrated with an US device to monitor applied pressure, demonstrating high sensitivity, repeatability, and reproducibility [305]. Probe tilt can also affect muscle and AT measurements by altering the angle of insonation, leading to anisotropy, an artifact in which tissue EI varies according to the angle between the US beam and the tissue fibers [306]. Although additional attachments have been used to monitor tilt [307], this can be passively monitored at landmarks with underlying bony surface by obtaining a strong bone echo (Figure 10).

Grayscale-based US imaging parameters can vary significantly between devices, and if adjusted, can drastically alter analysis of muscle EI and other composition-related metrics. Muscle EI is influenced not only by the device’s software version, resolution and transducer type, but also by image quality settings such as gain, focus, time-gain compensation, and imaging depth [269,308,309], as well as the ROI size, shape, and positioning [310]. To ensure comparability between individuals and across follow-up assessments, image quality settings and ROI parameters must be standardized, consistently maintained and reported, as well as any other manufacturer/model-specific algorithms included in the device that can be altered with software updates. If US software versions differ, cross-calibration equations can be developed using an US phantom.

#### Data presentation

Clear and systematic reporting of the methodology for US assessments is essential to enable comparability between studies, reproducibility of methods, and evaluation of the findings’ reliability. Aspects that should be reported by authors when describing data acquisition, analysis and verification methods are listed in Box 9.

BOX 9 **Essential reporting elements for publications involving body composition assessment by US**

• Participant characteristics, including sex, age, race/ethnicity, health status, and likelihood of experiencing edema or ascites.
• US device mode, model, and manufacturer.
• Transducer type and frequency.
• Device settings (relevant to the evaluated measurement, including gain, focus, time-gain compensation, and imaging depth), and whether/which were held constant or adjusted across image acquisition (within and between participants).
• Number of evaluators and their experience.
• Participant positioning, including limb placement and tension level (i.e., relaxed, contraction maneuvers), and duration spent in the final position to allow fluid equilibrium, when applicable, during anatomical landmarking and image acquisition.
• Detailed description of the anatomical landmarks adopted for each evaluation site, including the reference(s) on which they were previously adopted and/or validated.
• Adopted landmarking strategy (e.g., permanent markers) and chosen body side (i.e., right/left, dominant/nondominant), when applicable.
• Evaluated measurements and specific tissues/layers/muscles assessed.
• Level of compression applied to the transducer during image acquisition, along with the strategies used to verify target compression.
• Anatomical plane and inclination of the transducer during image acquisition.
• Number of acquired images per site and adopted analytical method to summarize them (e.g., mean).
• Image analysis protocol, including a description of the adopted visual landmarks and caliper inclination/positioning for measurements, and whether they were performed using built-in US software or post collection using external computer-aided software (provide name, version, and manufacturer).
• Intra- and inter-rater (when applicable) coefficient of variation assessment strategy for landmarking, image acquisition, and image analysis/data extraction (including, when applicable, evaluator blinding, whether the landmark was re-located, and interval between assessments).
• Consider providing (as a manuscript figure) one of the US images, marked with measurement calipers, to illustrate the adopted method for image acquisition and measurements/data extraction.

Abbreviation: US, ultrasound.

Alt-text: BOX 9

Although raw muscle and AT metrics are important for interpretation, they have also frequently been normalized to measures of body size, including BMI, height, body mass, and limb length [311]. Given the known sex-related differences in body composition and anthropometrics, further considerations are required to determine the relative importance of normalized and raw US metrics of muscle and AT for males and females separately. With the absence of well-standardized approaches, the reporting of results presenting both absolute and, when applicable, relative metrics (stratified by sex), facilitates comprehensive interpretation and enables comparisons across studies.

### Comparing findings across US devices

Several US manufacturers and probe types are available within clinical and research settings, with studies showing high reliability across models for size [312,313] and architectural [314] measurements. Even handheld B-mode US devices are emerging as useful tools, providing similar measures of MT as those obtained using clinical models [315]. However, as noted above, measurements of muscle composition (e.g., mean EI) vary significantly across devices [316] and parameter settings [317], limiting comparisons with studies that implement the same model and transducer. Although cross-device correction factors for muscle EI evaluation have been previously proposed [318], there has been limited uptake, likely due to the lack of commercially available standardized phantoms.

### Selecting an appropriate US device

When selecting an US device for research or clinical use, it is critical to consider the imaging needs (e.g., imaging mode), accessibility (e.g., costs), portability (e.g., cart compared with handheld), software enhancements (e.g., panoramic imaging), and types of transducers (e.g., linear, curvilinear/convex, frequency ranges). For instance, the B-mode US is generally preferred over the Amplitude-mode (A-mode) because it offers a more comprehensive assessment of body composition. In contrast, A-mode uses a basic, 1-dimensional approach that displays tissue depth from a single-pulse sound wave over time. This US mode only provides information on tissue thickness and does not allow for the evaluation of muscle composition or macroarchitectural properties. Linear transducers are the most suitable and versatile choice for most US measurements of skeletal muscle and AT, whereas the utility of curvilinear/convex transducers is restricted to specific assessments, such as intra-abdominal AT thickness and CSA of larger muscles. Additionally, a transducer with a wide frequency range offers further imaging options, as the resolution and penetration depth of the sound waves emitted by the probe is determined by its frequency. Although the penetration depth decreases at higher frequencies, the resolution increases, and vice versa. High-frequency (7–12 MHz) linear transducers are typically used to assess superficial tissues, such as most muscle sites and SAT, whereas low-frequency (2–5 MHz) curvilinear transducers are better suited for measuring intra-abdominal AT thickness.

Although most clinical US devices are able to provide basic muscle and AT thickness metrics, some features can enhance measurement capabilities. For example, in muscles with larger CSAs (e.g., vastus lateralis), US devices with imaging features such as panoramic imaging may provide a means of capturing the lateral fascial borders for these muscles, enabling proper analysis of the whole-muscle area [242]. Panoramic imaging also enables the entire FL to be captured within the US field of view. When this feature is unavailable, FL can instead be estimated by extrapolating the fascicle to its intersection with the superficial aponeurosis, as shown in Figure 8. Regarding muscle composition, most metrics are not comparable across devices and transducers. Thus, the availability of specific corresponding normative data may be an important consideration when choosing a device for such assessments. However, the limited number of studies that have attempted to establish such data are constrained by methodological limitations, including suboptimal diagnostic accuracy, small sample sizes after sex and age stratifications, and challenges in standardizing the depth of muscle EI measurements across individuals [239,[319], [320], [321]].

### Special considerations for US in specific populations/conditions

#### Obesity and rapid weight changes

Palpation and identification of surface anatomical sites during landmarking for US measurements can be challenging in individuals with abdominal obesity, particularly for trunk landmarks such as the anterior superior iliac spine. However, these challenges have not been formally quantified for reliability in the context of excess body weight, making it unclear to what degree these factors negatively influence the landmarking process. Practical approaches include asking individuals to palpate and identify their own bony landmarks (with evaluator verification); using an US probe to locate the bony landmarks of interest, an approach that requires substantial anatomical knowledge but becomes feasible with sufficient experience; or alternatively, relying on the evaluator’s anatomical knowledge. Moreover, increased SAT thickness attenuates US beams in deeper tissues, potentially reducing the visual distinction of muscle fascia borders. This can challenge the analysis of MT and muscle CSA, leading to greater variability in these measurements. The SAT layer can also interfere with the interpretation of mean EI (i.e., higher SAT thickness may result in lower EI because of beam scattering and reflection), and this effect is more pronounced in individuals with obesity [322,323]. Future research should aim to identify approaches to improve image resolution and muscle EI assessment in individuals with obesity, including investigations into the potential influence of SAT thickness on the reliability of serial EI measurements.

#### Hydration status and edema

Patients who are critically ill present with mild to severe hydration changes and fluid shifts [287,294]. Although muscle wasting can be detected in edematous patients [294], the clinical impact of fluid retention on US measurements of skeletal muscle is not yet fully understood. In a landmark study, Puthucheary et al. [241] demonstrated ∼10% loss of rectus femoris CSA by day 7; however, the rate of decline observed in muscle fiber CSA (obtained from muscle biopsies) displayed ∼18% reduction. The greater loss of fiber CSA compared with whole-muscle area may be due to accumulation of interstitial fluid (i.e., edema) within critically ill patients, which artificially inflates US-derived muscle measurements and obscures the true degree of atrophy. Supporting this, Fischer et al. [324] reported that MT of the quadriceps increased in cardiothoracic surgery patients from preoperation to postoperation by ∼0.4 cm, which was correlated with cumulative fluid balance during the first 3 postoperative days, likely due to interstitial fluid accumulation. Despite this increased MT after surgery, at hospital discharge, these patients still presented with ∼0.3 cm lower MTs compared with the preoperative baseline, highlighting the degree of muscle atrophy experienced in an acute stay. Future work closely monitoring fluid balance and edema is needed to understand how accumulation of fluids influences such assessments (likely relevant to all imaging modalities, not just US). These findings suggest that fluid retention may lead to overestimation of muscle size by US, particularly in the early postoperative or acute phases.

To attenuate the influence of fluid accumulation on US assessments, some authors have proposed the use of maximal compression of the transducer against the skin and its underlying tissue layers [271,278]. In healthy young and older adults, minimal (compared with maximal) compression of the quadriceps muscle layer thickness resulted in stronger correlations with ALST from DXA [252], favoring the use of minimal compression. However, in critically ill patients, maximal compression provided slightly stronger associations with CT-based L3 CSA compared with minimal compression [325]. Future work is needed to clarify the relative role of tissue compression of measures of muscle mass across diverse populations with and without fluid accumulations. However, standardization for maximal compression is challenging, as different users and probe footprints can lead to differing degrees of tissue deformation. Moreover, most studies utilize minimal compression for thickness, CSA, and composition analyses; hence we recommend its use for generalizability (Figure 10).

#### Other conditions

US-based body composition assessments can be difficult and potentially inaccurate in patients with conditions such as stroke, where one side is paretic, or in those with trauma at the intended evaluation site. Some degree of asymmetry between left and right or dominant and nondominant muscles is expected; however, in healthy individuals, these differences are generally small and not considered clinically significant [253,279]. In the clinical scenarios mentioned above, similar results for both sides cannot be assumed [276], and the evaluation of both the affected and unaffected side (particularly for longitudinal assessments) is recommended.

### Final remarks and perspectives

This second publication in our series on methodological standards provides practical guidance on standardized terminology, assessment, and reporting for commonly used body composition methods. By addressing key principles, validity, and procedural considerations, we aim to promote consistency across research and clinical practice. We acknowledge that some recommendations may be difficult to implement in settings with limited infrastructure or resources (e.g., device or system cross-calibration or reliability testing). However, guidance related to terminology, assessment procedures, data reporting, and population-specific applications is broadly applicable across diverse contexts. Moreover, the methods reviewed (bioelectrical impedance approaches, DXA, CT, and US) are among the most widely used globally and align with existing clinical guidelines for body composition assessment [[7], [8], [9], [10], [11]].

Future publications in the series will cover additional methods, including ADP, MRI, MRS, and emerging methods such as D3-creatine dilution, TBW, and 3-dimensional optical scanning. We hope to also address additional topics such as clinical applications, including population-specific considerations and the role of body composition in guiding decision-making. Selected topics, such as PhA and a more in-depth discussion of MDC, are also planned. As the field continues to evolve, artificial intelligence is playing an increasingly prominent role, particularly in enabling automated analysis. Accordingly, future work will explore the validity, limitations, and broader applications of artificial intelligence to improve precision, efficiency, and scalability of body composition assessment.

## Author contributions

The authors’ responsibilities were as follows – CMP, MCG, KN, SBH: designed and led the group; CEO: was the project coordinator; and all authors: were responsible for co-writing the manuscript and have reviewed and approved the final manuscript.

## Endorsements

We are seeking endorsements from clinical nutrition societies and affiliated journals to promote consistency and adherence to these standards.

## Data availability

Data described in the manuscript, codebook, and analytic code will be made available upon request pending application and approval.

## Funding

CMP is partially supported by the Canada Research Chairs Program. MCG is partially supported by the Brazilian National Council for Scientific and Technological Development (CNPq, Brazil). The funders had no role in the project design, interpretation of evidence, and writing of the manuscript.

## Declaration of Generative AI and AI-assisted technologies in the writing process

The authors declare that no generative AI or AI-assisted technologies were used in the writing of this manuscript.

## Conflict of interest

CMP has received honoraria and/or paid consultancy from Abbott Nutrition, Nutricia, Nestlé Health Science, and Novo Nordisk. MCG reports receiving speaking fees from Abbott Nutrition, Danone/Nutricia, and Nestlé Health Science Brazil. CEO reports honoraria and speaking fees from Abbott Nutrition. TC reports speaking fees from Nutricia, Fresenius Kabi, Nestlé and Abbott. OTC has received research support from Eli Lilly and Co, Nestlé, and Novo Nordisk. LG has received paid consultancy and speaking honoraria by Fresenius Kabi, paid consultancy by Fresenius Kabi and Eli Lilly, and research support from Nestlé Health Science. SBH serves on the Medical Advisory Boards of Tanita Corporation, Novo Nordisk, Lilly, Abbott, Regeneron, and Medifast. All other authors report no conflicts of interest.

## Appendix A Supplementary data

Multimedia component 1 Supplemental Tables and Boxes
