# Supplementary material for: Methodological standards for body composition assessment—an expert-endorsed guide for research and clinical applications: bioimpedance, dual-energy X-ray absorptiometry, computerized tomography, and ultrasound methods
Source: Am J Clin Nutr. 2026 Mar 19;123(5):101283. doi: 10.1016/j.ajcnut.2026.101283 (PMC13197919; doi:10.1016/j.ajcnut.2026.101283)
Supplement: Multimedia component 1 [file mmc1.pdf]

Methodological Standards for Body Composition Assessment - an Expert-Endorsed Guide for Research and Clinical Applications: Bioimpedance, Dual-energy X-ray Absorptiometry, Computerized Tomography, and Ultrasound Methods

Prado CM et al.

Supplemental Table 1. Glossary of body composition terms and suggested abbreviations…………………………………………………………………… 2
Supplemental Table 2. Key terminology for measurement validity…………….. 6
Supplemental Table 3. Common myths and best practices in bioimpedance assessment ……………………………………………………………………... 7
Supplemental Box 1. Best-use scenarios for bioimpedance-based body composition assessment……………………………………………………....... 8
Supplemental Box 2. Common pitfalls in body composition assessment using bioimpedance……………………………………………………………………... 9
Supplemental Box 3. What not to do when using bioimpedance for body composition assessment (DON'Ts)……………………………………………. 10
Supplemental Box 4. Research gaps in body composition assessment using bioimpedance……………………………………………………………………... 11
Supplemental Box 5. Best-use scenarios for dual-energy X-ray absorptiometry (DXA)-based body composition assessment………………………………….... 12
Supplemental Box 6. Common pitfalls in body composition assessment using dual-energy X-ray absorptiometry (DXA)………………………………………….. 13
Supplemental Box 7. What not to do when using dual-energy X-ray absorptiometry (DXA) for body composition assessment (DON'Ts)………………. 14
Supplemental Box 8. Research gaps in body composition assessment using dual-energy X-ray absorptiometry (DXA)………………………………………….... 15
Supplemental Box 9. Best-use scenarios for body composition assessment using computerized tomography (CT)……………………………………………………... 16
Supplemental Box 10. Common pitfalls in body composition assessment using computerized tomography (CT)……………………………………………………... 17
Supplemental Box 11. What not to do when using computerized tomography (CT) for body composition assessment (DON'Ts)………………………………….. 18
Supplemental Box 12. Research gaps in body composition assessment using computerized tomography (CT)……………………………………………………... 19
Supplemental Box 13. Best-use scenarios for ultrasound (US)-based body composition assessment……………………………………………………………... 20
Supplemental Box 14. Common pitfalls in body composition assessment using ultrasound (US)……………………………………………………………………… 21
Supplemental Box 15. What not to do when using ultrasound (US) for body composition assessment (DON'Ts)……………………………………………….. 22
Supplemental Box 16. Research gaps in body composition assessment using ultrasound (US)……………………………………………………………………… 23

Supplemental Table 1. Glossary of body composition terms and suggested abbreviations.

**Recommended terminology**	**Rationale/Definition**
Body composition/components terms	
AT	AT is classified as a type of connective tissue composed of adipocytes, collagen, elastic fibers, fibroblasts, capillaries, and ECF.<br>About 80% of AT is made up of storage triglycerides (fat), that are part of the body's energy reserve.<br>AT includes multiple cell types and is distributed in several subdivisions (1), such as SAT, VAT, IMAT, and intraMAT. Intramyocellular lipids are present within skeletal muscles and consist of an outer phospholipid layer and inner triglyceride core. Intracellular fat is also present in hepatocytes (liver cells).
Fat	"Fat" generally refers to esters of fatty acids, or a mixture of such compounds, that occur in living beings. The term refers specifically to triglycerides (triple esters of glycerol) that are the main non-polar or "neutral" lipids components of fatty tissues in animals (2).<br>Fat is the major contributor to total body lipids; the remaining lipids are components of the FFM (3).
Total lipids	Total lipids are carbon- and hydrogen-containing compounds that are relatively insoluble in water and soluble in solvents such as ether, benzene, and alcohol (4). The complex lipids found in biological systems include polar lipids (also termed as "non-fat lipids") and non-polar lipids (also termed as "fat'). Polar lipids consist of phospholipids and glycolipids that function in cell membranes and perform other vital cell functions. Non-polar or "neutral" lipids are predominantly triglycerides, a long-term energy source (5).
IMAT	IMAT refers to adipocytes found in small clusters and as individual cells between muscle fascicles and muscle groups (6).

**Recommended terminology**	**Rationale/Definition**
IntraMAT	IntraMAT refers to adipocytes located within muscles but outside muscle fibers, accounting for the EMCL detected and visualized using magnetic resonance spectroscopy.
Skeletal muscle	Skeletal muscle is composed of muscle cells, connective tissues, and other components; when measured *in vivo,* excludes embedded AT and fat cells.
FFM	Everything in the body, excluding fat (triglycerides). FFM contains nonfat lipids (essential or structural lipids).<br>LBM is not a clearly defined chemical component, and for that reason, early leaders in body composition research agreed to no longer use this term in scientific publications (7).<br>“Lean mass” is acceptable as a synonym for FFM but should not be confused with LST. Unfortunately, DXA reports incorrectly label LST as lean mass, leading to a common terminological mistake.
LST	LST is the difference between body weight and the sum of fat and BMC, and includes nonfat lipids. LST is a term generally specific to DXA, given DXAs ability to quantify the following 3 components: fat (usually calibrated against triglyceride standards), BMC (the mineral portion of bone), and LST.<br>LST includes 6 regional components: both arms, both legs, trunk, and head. The LST sum of all 4 extremities is referred to as ALST. ALM is the sum of ALST and BMC.
TBP	TBP, found within FFM and LST, includes the intracellular and extracellular proteins across the body’s organs and tissues.
BCM	This component represents all the body’s cell mass, including ICF and intracellular solids.
TBW	TBW is a major molecular-level component present in the FFM portion of body mass. TBW is distributed in the ICW and ECW spaces.

**Recommended terminology**	**Rationale/Definition**
ECF	Fluids found in the extracellular space, including ECW, minerals/electrolytes, circulating proteins, and other substrates.
ECS	The remainder of body mass after subtracting BCM and ECF.
BMC	BMC is the mineral portion of bone.
Technical terms	
Bioimpedance methods	SF-BIA, MF-BIA, and BIS differ technically; use "bioimpedance methods" when referring to all, and "BIA" only for SF-BIA and/or MF-BIA.
DXA	DXA is preferred based on consensus and consistency with predecessor technology (DPA).
Ultrasound	"Ultrasound" instead of "ultrasonography" is the preferred and most used term in clinical and research contexts.
Muscle composition	Use "muscle composition" when referring to muscle radiodensity in CT or muscle echo intensity in US. "Muscle quality" is a broader concept (structure, function, morphology, architecture).

Abbreviations: ALM, appendicular lean mass; ALST, appendicular lean soft tissue; AT, adipose tissue, BCM, body cell mass; BIS, bioimpedance spectroscopy; BMC, bone mineral content; CT, computerized tomography; DXA, dual-energy X-ray absorptiometry; ECF, extracellular fluids; ECS, extracellular solids; ECW, extracellular water; FFM, fat-free mass; ICW, intracellular water; ICF, intracellular fluids; IMAT, intermuscular adipose tissue; intraMAT, intramuscular adipose tissue; LBM, lean body mass; LST, lean soft tissue; MF-BIA, multifrequency bioelectrical impedance analysis; SAT, subcutaneous adipose tissue; SF-BIA, single-frequency bioelectrical impedance analysis; TBP, total body protein; TBW, total body water; US, ultrasound; VAT, visceral adipose tissue.

Reference:

1. Shen W, Wang Z, Punyanita M, Lei J, Sinav A, Kral JG, et al. Adipose tissue quantification by imaging methods: a proposed classification. Obes Res. 2003;11(1):5-16.
2. Biology Dctionary. Fat. 2017 [Available from: https://biologydictionary.net/fat/]. Accessed 26th Jan 2024.
3. Comizio R, Pietrobelli A, Tan YX, Wang Z, Withers RT, Heymsfield SB, et al. Total body lipid and triglyceride response to energy deficit: relevance to body composition models. Am J Physiol. 1998;274(5):E860-6.

4. Fahy E, Subramaniam S, Brown HA, Glass CK, Merrill AH, Jr., Murphy RC, et al. A comprehensive classification system for lipids. J Lipid Res. 2005;46(5):839-61.
5. Behnke AR, Osserman EF, Welham WC. Lean body mass; its clinical significance and estimation from excess fat and total body water determinations. AMA Arch Intern Med. 1953;91(5):585-601.
6. Goodpaster BH, Bergman BC, Brennan AM, Sparks LM. Intermuscular adipose tissue in metabolic disease. Nat Rev Endocrinol. 2023;19(5):285-98.
7. Fidanza F. Body fat in adult man: semicentenary of fat density and skinfolds. Acta Diabetol. 2003;40 Suppl 1:S242-5.

Supplemental Table 2. Key terminology for measurement validity.

Concept	Definition
Validity	Overall degree to which a method measures what it is intended to measure; encompasses accuracy, precision, and bias.
Accuracy	The extent to which an index test agrees with a reference standard (more established method).
Precision	The degree of agreement between repeated measurements (within or between assessors).
Reliability	High repeatability and reproducibility, or the variability within or between assessors is minimal.
MDC	The smallest change that exceeds measurement error and reflects a true change. MDC thresholds are method-, device-, and protocol-dependent and should ideally be derived from test-retest precision using the specific acquisition and analysis protocol employed.

MDC, minimal detectable change.
Reference: Earthman CP. Body composition tools for assessment of adult malnutrition at the bedside: a tutorial on research considerations and clinical applications. *JPEN J Parenter Enteral Nutr.* 2015;39(7):787-822.

Supplemental Table 3. Common myths and best practices in bioimpedance assessment.

Myth	Reality	Best practice
Bioimpedance directly measures fat and muscle.	Bioimpedance reflects body water volume and distribution; body composition is derived indirectly through prediction equations.	Consider raw measurements as primary data; interpret body composition as model outputs.
More decimal places in output means greater accuracy.	Display precision differs from accuracy or repeatability.	Define meaningful change using test-retest error.
Devices, modes, or equations are interchangeable.	Switching devices or equations can create artificial changes.	Maintain strict measurement invariance.
Indices such as "visceral fat rating," "metabolic age," or "body type" are directly measured quantities.	These are algorithm-derived indices that may lack transparent validation.	Do not equate proprietary indices with imaging-derived biomarkers unless validated.
Phase angle is a universal cell health score.	Phase angle is influenced by age, sex, hydration, and disease.	Interpret using appropriate reference standards.
Electrode placement and posture are minor details.	Small deviations significantly affect impedance values.	Standardize and document measurement conditions.
A change in body composition reflects an underlying change in physiology.	Bioimpedance outputs are dependent on many factors.	Assess protocol adherence before concluding true body composition change.

**Supplemental Box 1.** Best-use scenarios for bioimpedance-based body composition assessment.
• When individuals are well matched to validated prediction equations, falling within the characteristics of the equation development sample.<br>• Longitudinal monitoring of individuals under standardized conditions, with established minimal detectable change values.<br>• When access to advanced imaging is limited or in large population studies, where low cost, portability, and rapid assessment support high-throughput data collection.<br>• When a non-invasive, radiation-free method is needed for repeated assessments for clinical screening and routine monitoring.

Supplemental Box 2. Common pitfalls in body composition assessment using bioimpedance.

- Applying prediction equations outside their development population reduces accuracy, especially in individuals with different body size/shape or clinical characteristics.
- Relying on devices that use undisclosed, proprietary prediction equations limits transparency and leads to inconsistent body composition estimates across systems.
- Assuming interchangeability is incorrect: results from different devices should not be compared without proper cross-validation.
- Ignoring hydration status and timing effects (e.g., diurnal fluid shifts, recent food/fluid intake, edema, ascites) compromises reliability and validity.
- Violating core bioimpedance assumptions (uniform cylinder body shape, stable fluid distribution), as occurs in obesity, pregnancy, and fluid-overload states, biases estimates.
- Metallic implants and silicone breast implants may bias bioimpedance estimates, although evidence remains limited.
- Overlooking multiple sources of measurement error (impedance, prediction equation, reference method, modeling, and biological variability) leads to overconfidence in individual-level results.

**Supplemental Box 3.** What not to do when using bioimpedance for body composition assessment (DON'Ts).
• Do not assume bioimpedance directly measures body composition. It estimates components using device- and population-specific equations or biophysical modeling.<br>• Do not assume prediction equations remain valid outside their development population (e.g., obesity, pregnancy, or clinical states with altered body shape/fluid distribution). In these groups, interpret estimates cautiously and prioritize standardized longitudinal monitoring when used.<br>• Do not treat devices, brands, or software as interchangeable; cross-device/software comparisons require formal cross-validation.<br>• Do not rely on "black-box" devices without understanding the underlying equations, assumptions, and limitations.<br>• Do not ignore hydration, timing, or testing conditions; fluid shifts, food/water intakes, exercise, edema/ascites, and circadian variation can bias results.<br>• Do not overlook multiple sources of error (measurement, regression, modeling, reference method, and biological variability), particularly at the individual level.<br>• Do not assume accuracy in special populations without evidence, including individuals with large or centrally located metallic implants.

**Supplemental Box 4.** Research gaps in body composition assessment using bioimpedance.
• Prediction equations are insufficiently validated in many clinical populations, restricting generalizability beyond healthy or reference samples.<br>• Minimal detectable change values are lacking, limiting study design, power calculations, and interpretation of longitudinal change.<br>• The influence of metallic implants and silicone breast implants and environmental and physiological factors (e.g., skin temperature) on measurements is not well characterized, and strategies to minimize these effects are needed.<br>• Evidence for the validity of bioimpedance spectroscopy in patients undergoing dialysis is inconsistent and requires further investigation.<br>• Limited evidence on workflow, cost-effectiveness, and implementation in routine care.

**Supplemental Box 5.** Best-use scenarios for dual-energy X-ray absorptiometry (DXA)-based body composition assessment.
• When precise regional and whole-body composition is required, including fat mass, lean soft tissue or fat-free mass, and bone mineral content.<br>• For risk stratification and prognostic assessment, including identification of low muscle mass, altered fat distribution, osteoporosis, or their combination.<br>• For longitudinal monitoring of body composition change, particularly in interventions, disease progression, or recovery, when standardized protocols are maintained and scanner is available.

**Supplemental Box 6.** Common pitfalls in body composition assessment using dual-energy X-ray absorptiometry (DXA).
• Misinterpreting output terminology, since definitions of "lean," "lean soft tissue," "fat-free mass," and "lean + bone mineral content" differ by manufacturer and software version.<br>• Changing scan or analysis settings across timepoints, which alters software calculations and compromises longitudinal comparability.<br>• Assuming interchangeability across systems, despite known differences between DXA manufacturers, models, and software that affect pooled or multi-center analyses.<br>• Ignoring body size, positioning, and anatomical features (e.g., excess breast tissue, anatomical variants, asymmetry), which can bias regional estimates.<br>• Not accounting for fluid shifts and non-biological materials; edema, ascites, recent exercise, implants, casts, and other objects are incorporated into lean soft tissue/fat-free mass or interfere with X-ray attenuation.<br>• Overlooking regulatory and ethical restrictions, as eligibility for DXA varies by country, population, and clinical indication.<br>• Failing to apply appropriate safety screening, including avoiding DXA in pregnancy and assessing contraindications.

Supplemental Box 7. What not to do when using dual-energy X-ray absorptiometry (DXA) for body composition assessment (DON'Ts).

- Do not interpret changes of <3% in fat mass and of <2% in lean soft tissue as meaningful, as they are within measurement error and may not be reliably detected.
- Do not assume output terminology is consistent across systems.
- Do not change scan settings, positioning, or analysis protocols across timepoints, as this compromises longitudinal comparability.
- Do not treat data from different DXA manufacturers, models, or software versions as interchangeable without cross-calibration.
- Do not overlook body size limits, positioning artifacts, or anatomical features (e.g., excess breast tissue, asymmetry) that can bias regional estimates.
- Do not ignore fluid shifts or non-biological materials; edema, ascites, recent exercise, implants, casts, and other objects can bias lean soft tissue/fat-free mass estimates and X-ray attenuation.
- Do not use hemiscans when meaningful left-right asymmetry is present for whole-body composition estimates.
- Do not ignore regulatory and ethical restrictions; eligibility varies by country, population, and clinical indication.
- Do not scan pregnant individuals and always perform appropriate pregnancy screening when indicated.

**Supplemental Box 8.** Research gaps in body composition assessment using dual-energy X-ray absorptiometry (DXA).
• The impact of excess breast tissue on appendicular lean soft tissue remains poorly quantified, and standardized strategies to minimize this bias are lacking.<br>• The influence of altered hydration and fluid accumulation (e.g., edema, ascites) on longitudinal body composition is not well established, particularly in clinical populations.<br>• The effects of metal-artifact imputation on soft tissue estimates are unclear; while bone measures are known to be affected, the impact on lean soft tissue and fat mass remains controversial.<br>• Existing whole-body phantoms lack accuracy, limiting their use for cross-calibration when in vivo methods are not feasible.

Supplemental Box 9. Best-use scenarios for body composition assessment using computerized tomography (CT).

- When CT scans are already acquired for diagnosis, staging, or treatment monitoring (i.e., oncology and other chronic disease), avoiding additional radiation exposure and cost.
- As a prognostic and risk stratification tool to characterize muscle and adipose tissue phenotypes associated with adverse clinical outcomes (e.g., low muscle mass, myosteatosis, and abnormal adipose tissue distribution) and to support identification of sarcopenia/sarcopenic obesity in conjunction with other assessments.
- When longitudinal clinical scans are available, allowing within-individual comparisons across time points, particularly to detect moderate-to-large changes.
- As a reference standard for validating other body composition methods or automated algorithms.
- When appropriate quality assurance procedures and calibration phantoms are used, CT provides high accuracy and reliability.

**Supplemental Box 10.** Common pitfalls in body composition assessment using computerized tomography (CT).
• Mostly restricted to clinical populations due to radiation exposure; not widely available in healthy individuals. Weight and bore limits exclude some individuals.<br>• Longitudinal analyses depend on whether follow-up scans exist in medical records.<br>• Automated methods require strict quality control to avoid low quality segmentations.<br>• Reproducibility assessments rarely include repeat scanning, limiting reliability estimates.<br>• A single slice or muscle group may not reflect regional or whole-body changes.<br>• Motion, fluid overload/retention, metal implants, or monitoring leads can degrade image quality and contribute to missing data. High body mass increases image noise and positioning errors.<br>• Contrast use and tube voltage alter tissue radiodensity and can affect body-composition measures.

Supplemental Box 11. What not to do when using computerized tomography (CT) for body composition assessment (DON'Ts).

- Do not assume CT is feasible for all individuals; scanner weight and bore size limits, and pregnancy may exclude individuals.
- Do not assume longitudinal CT data will be available for all individuals, as repeat imaging is determined by clinical care rather than research needs.
- Do not assume observer experience compensates for poor slice selection; slice selection is a major source of measurement error.
- Do not accept semi- or fully automated segmentation outputs without a formal quality-control procedure.
- Do not rely on a single slice or single muscle group when detecting small longitudinal changes or inferring whole-body composition.
- Do not compare/mix scans acquired with different acquisition protocols (e.g., tube voltage, contrast use, or contrast phase) without appropriate standardization.
- Do not neglect cross-calibration when using different CT scanners or acquisition protocols.
- Do not rely on contrast-enhanced scans for radiodensity quantification; reserve them only for tissue segmentation.
- Do not include low-quality images affected by motion, metal artifacts, excess image noise, or fluid overload/retention.

Supplemental Box 12. Research gaps in body composition assessment using computerized tomography (CT).

- Reliability estimates are limited because repeat scanning is rarely performed.
- The precision of longitudinal change from serial CT scans remains uncertain.
- Minimal detectable change has not been systematically summarized, limiting design and sample-size planning.
- There is limited consensus on definitions and distinctions between intermuscular adipose tissue and intramuscular adipose tissue.
- It remains uncertain whether multiple slices outperform a single cross-sectional area for capturing regional or whole-body composition change.
- Adipose tissue radiodensity is emerging metric but requires further validation.
- Evidence on workflow, cost-effectiveness, and implementation in routine care is limited.

Supplemental Box 13. Best-use scenarios for ultrasound (US)-based body composition assessment.

- For point-of-care and bedside evaluation, including in critically ill, hospitalized, or immobile patients where transport to imaging facilities is not feasible.
- For longitudinal monitoring of regional muscle and adipose tissue change (e.g., during interventions, rehabilitation, disease progression, or recovery), when standardized landmarks and protocols are maintained.
- When targeted, site-specific assessment is clinically meaningful, including measurement of muscle thickness and cross-sectional area, and subcutaneous adipose tissue thickness at predefined anatomical landmarks.
- For evaluation of muscle composition, using surrogate metrics (e.g., echo intensity) to assess non-contractile tissue infiltration and relate structure to functional or metabolic outcomes.
- In settings with limited access to advanced imaging modalities, where cost, infrastructure, or availability restrict their use.

**Supplemental Box 14.** Common pitfalls in body composition assessment using ultrasound (US).
• Measurement of reliability varies widely due to differences in training and experience, landmarking, probe compression/tilt, and image acquisition/analysis. Few studies have evaluated reliability of the full protocol, including re-landmarking.<br>• Image acquisition time can take up to ~15 minutes but varies with operator experience, number of sites assessed, repeated measurements, and subject-specific factors.<br>• The absence of globally accepted imaging and analysis standards limits cross-study comparability and the development of normative reference data.<br>• US cannot distinguish contractile tissue from intramuscular adipose tissue and connective tissue; muscle size measures therefore include non-contractile tissue.<br>• Most commercial probes capture only narrow, superficial regions, constraining cross-sectional area assessments to smaller muscles and limiting adipose assessment to tissue thickness rather than volumetric measures.<br>• US settings (e.g., gain, depth, focus), transducer type, software version, and region of interest definition can substantially alter muscle echo intensity, limiting cross-study comparisons.<br>• Thick subcutaneous adipose tissue attenuates the ultrasound beam, obscuring muscle borders, increasing variability in muscle thickness/cross-sectional area, and biasing muscle echo intensity. Edema and inflammation can also confound echo intensity.<br>• Accurate landmarking is challenging in abdominal obesity, paretic limbs, trauma, or localized pathology, and anterior-only landmarks may not represent whole-muscle composition.

**Supplemental Box 15.** What not to do when using ultrasound (US) for body composition assessment (DON'Ts).
• Do not assume reliability is inherent; intra- and inter-rater reliability varies and must be tested and reported, including re-landmarking.<br>• Do not change landmarks, probe pressure/tilt, positioning, or analysis protocols across timepoints, as this compromises longitudinal comparability.<br>• Do not interpret muscle thickness or cross-sectional area as purely contractile tissue; US cannot separate intramuscular adipose and connective tissues from muscle, and size metrics include non-contractile components.<br>• Do not treat muscle echo intensity as adipose infiltration alone; echo intensity is also influenced by fibrosis, edema, inflammation, and device settings.<br>• Do not ignore thick subcutaneous adipose tissue, as it attenuates the beam, obscures fascial borders, increases variability, and biases muscle echo intensity, particularly in individuals with obesity.<br>• Do not assume data are comparable across devices or settings; changes in gain, depth, focus, transducer type, software version, or regions of interest placement can substantially alter results.<br>• Do not assume prediction equations remain valid outside their development population (e.g., obesity, pregnancy, or clinical states with altered body shape/fluid distribution), reference standard, and imaging protocols. In these groups, interpret estimates cautiously and prioritize standardized longitudinal monitoring when used.<br>• Do not assume all sites or patients are appropriate; landmarking is challenging in abdominal obesity, and measurements may be unreliable in trauma, paresis, or other localized pathology.

**Supplemental Box 16.** Research gaps in body composition assessment using ultrasound (US).
• The independent effects of edema, inflammation, and necrosis on muscle echo intensity remain poorly quantified, particularly across aging and disease states.<br>• Beam attenuation by subcutaneous adipose tissue complicates comparisons of muscle echo intensity across individuals. Correction methods exist but are device-, population-, and muscle-specific and lack generalizability. Strategies to improve image resolution and the reliability of muscle echo intensity in individuals with obesity therefore require further investigation.<br>• Day-to-day repeatability of the full protocol, including re-landmarking, has not been adequately evaluated across diverse populations.<br>• Most existing prediction equations are derived from healthy adults; few are validated in clinical populations or establish how well single-muscle measures reflect whole-body musculature.<br>• Comprehensive protocols (e.g., 9-site approaches) improve body composition estimation but are often impractical for routine clinical use; streamlined yet valid alternatives are needed.<br>• Further work is needed to determine whether normalized versus raw metrics of muscle and adipose tissues should be interpreted differently in males and females.
