# Supplementary material for: Methodological standards for body composition assessment—an expert-endorsed guide for research and clinical applications: bioimpedance, dual-energy X-ray absorptiometry, computerized tomography, and ultrasound methods
Source: Am J Clin Nutr. 2026 Mar 19;123(5):101283. doi: 10.1016/j.ajcnut.2026.101283 (PMC13197919; doi:10.1016/j.ajcnut.2026.101283)
Supplement: Supplemental Figure 1 [file mmc2.pdf]

Methodological Standards for Body Composition Assessment - an Expert-Endorsed Guide for Research and Clinical Applications: Bioimpedance, Dual-energy X-ray Absorptiometry, Computerized Tomography, and Ultrasound Methods

Prado CM et al.

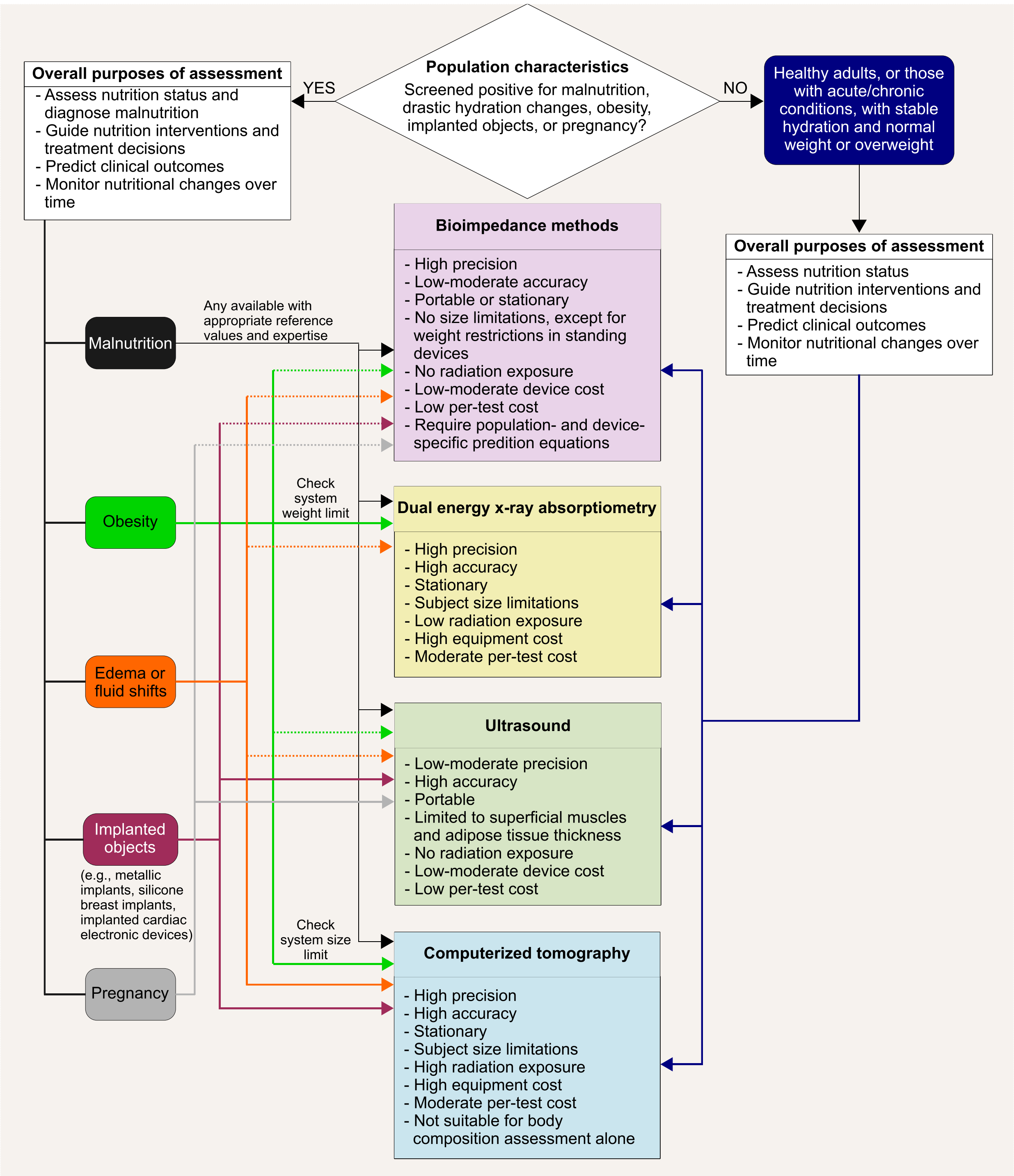

Supplemental Figure 1. Decision tree for selecting body composition assessment methods.

Muscle mass assessment methods were selected in accordance with the Global Leadership Initiative on Malnutrition (GLIM) guidelines. When malnutrition coexists with other conditions, follow the method-selection recommendations applicable to the predominant condition. For longitudinal monitoring, consider minimal detectable change values to determine whether observed changes exceed measurement error and

represent meaningful change. Dashed lines indicate methods with reduced accuracy or limited evidence for specific conditions (see text for details). Individuals with implanted devices may not be eligible for body composition assessment if the device lies within the field of assessment (see text for details). Healthy individuals may undergo single-slice computerized tomography (CT) for research purposes; similarly, patients with trauma or other acute conditions (e.g., kidney stones) who are otherwise healthy may have CT scans available in medical records that can be used for body composition assessment.
