# Supplementary material for: Methodological standards for body composition assessment—an expert-endorsed guide for research and clinical applications: bioimpedance, dual-energy X-ray absorptiometry, computerized tomography, and ultrasound methods
Source: Am J Clin Nutr. 2026 Mar 19;123(5):101283. doi: 10.1016/j.ajcnut.2026.101283 (PMC13197919; doi:10.1016/j.ajcnut.2026.101283)
Supplement: Supplemental Information 1 [file mmc3.pdf]

Methodological Standards for Body Composition Assessment - an Expert-Endorsed Guide for Research and Clinical Applications: Bioimpedance, Dual-energy X-ray Absorptiometry, Computerized Tomography, and Ultrasound Methods
Prado CM et al.

Body Composition Terminology & Reporting Checklist

(Based on AJCN Methodological Standards, 2025[1])

[1]Prado CM, Gonzalez MC, Norman K, Barazzoni R, Cederholm T, Compher C, Jensen GL, Abe T, Barbosa-Silva TG, Bosy-Westphal A, Carmichael OT, Earthman CP, Evans WJ, Fields DA, Genton L, Hu HH, Kara M, Miles-Chan JL, Mourtzakis M, Muller MJ, Orsso CE, Perkisas S, Sardinha LB, Shepherd JA, Siervo M, Strauss BJ, Yamada Y, Zhu S, Heymsfield SB. Methodological standards for body composition-an expert-endorsed guide for research and clinical applications: levels, models, and terminology. Am J Clin Nutr. 2025 122(2):384-391. doi: 10.1016/j.ajcnut.2025.05.022. PMID: 40754386.

Categories	Checklist Item
**Terminology Accuracy**	☐ Use standardized terms and definitions as outlined in the AJCN Methodological Standards manuscript[1]. For example:<br>• Fat-free mass (FFM) = all nonfat components (includes bone mineral content [BMC], nonfat lipids). FFM = Lean mass (LM)<br>• Lean soft tissue (LST) = all nonfat, non-bone components. LST = FFM minus BMC<br>• Skeletal muscle = tissue-organ level. Some methods (e.g., computerized tomography) can exclude embedded adipose tissue (AT)<br>• Fat mass (FM) = mainly triglycerides, assessed at the molecular level<br>• AT = use only when tissue-organ level is evaluated<br>☐ If method output uses non-standard terms, provide a clear definition and, if possible, correspondence to standard terms.<br>☐ Do NOT use 'lean body mass' (LBM) - replace with FFM or LST, as appropriate.<br>☐ Use LM only when generally referring to nonfat or non-adipose tissue components. This is the same as FFM.<br>☐ Clearly differentiate:<br>• FFM vs. LST vs. skeletal muscle<br>• FM vs. AT<br>☐ Use correct abbreviations (FFM, FM, LST, ALST, ALM, TBW, BCM, etc.).
**Methodological Details**	☐ Report device name, manufacturer, model, software version.<br>☐ Describe calibration procedures, if applicable.<br>☐ Identify reference method for prediction equations, if applicable.<br>☐ Note operator training and variability data (e.g., ICC), if available.<br>☐ Report and reference the data acquisition protocol and pre-assessment procedures, if applicable.
**Outcome Reporting**	☐ Present absolute values (kg) and indexed values (e.g., ALST/height^2) for overall group and stratified by sex.<br>☐ Use correct indexing terminology (e.g., 'appendicular lean soft tissue index').<br>☐ Avoid conflating outcomes from different levels (e.g., FM and AT, FFM and skeletal muscle).
**Historical Data & Literature Reviews**	☐ Map outdated terms to current standards when using older datasets or literature using outdated terminology.<br>☐ Document terminology harmonization in Methods.

ALM, appendicular lean mass; ALST, appendicular lean soft tissue; ICC, intraclass correlation coefficient; TBW, total body water
