# Supplementary material for: Methodological standards for body composition assessment—an expert-endorsed guide for research and clinical applications: bioimpedance, dual-energy X-ray absorptiometry, computerized tomography, and ultrasound methods
Source: Am J Clin Nutr. 2026 Mar 19;123(5):101283. doi: 10.1016/j.ajcnut.2026.101283 (PMC13197919; doi:10.1016/j.ajcnut.2026.101283)
Supplement: Multimedia component 4 [file mmc4.pdf]

Methodological Standards for Body Composition Assessment - an Expert-Endorsed Guide for Research and Clinical Applications: Bioimpedance, Dual-energy X-ray Absorptiometry, Computerized Tomography, and Ultrasound Methods

Prado CM et al.

Supplemental Information 2. Principles of muscle stiffness and skeletal muscle elastography.

Muscle stiffness is defined as the relation between compression and deformation (1). It is the combination of active tension produced by muscle contraction and passive tension produced by connective tissue such as collagen in the extracellular matrix. Deformation can be created either by external compression or by an intrinsic US push beam. The extent of tissue deformation is dependent on the elastic characteristics of the tissue. Shear is the change in the shape of tissues without a change in volume. As a result of the initial shear, neighboring tissue layers will undergo shear of their own, resulting in a wave. In skeletal muscle elastography, a shear wave is produced by manual compression or an acoustic radiation force. The distortion of the tissue is then mapped by sonography, and ultimately the wave is traced back to measure mechanical properties using algorithms. Stiffness can be measured by either displacement, strain or shear-wave velocity. Displacement is primarily measured in the axial direction. Strain is the deformation of tissue owing to stress. Stress is the ratio of applied force on the examined unit area and can be either longitudinal (i.e., perpendicular to the tissue) or shear (i.e., parallel to the tissue). Shear waves cause movement of particles perpendicular to the wave direction with a certain speed.

Reference:

1. Ryu J, Jeong WK. Current status of musculoskeletal application of shear wave elastography. Ultrasonography. 2017;36(3):185-97.
